# Anti-Inflammatory Effects of Acupuncture at ST36 Point: A Literature Review in Animal Studies

**DOI:** 10.3389/fimmu.2021.813748

**Published:** 2022-01-12

**Authors:** Ji-Eun Oh, Seung-Nam Kim

**Affiliations:** College of Korean Medicine, Dongguk University, Goyang, South Korea

**Keywords:** anti-inflammatory effects, acupuncture, acupoint ST36, literature review, animal study

## Abstract

So far, a number of acupuncture studies have shown anti-inflammatory effects of acupuncture treatment, mostly known at specific point ST36. However, there is no literature that oversaw the inflammation-regulatory effects of acupuncture in each tissue. Therefore, we investigated how acupuncture at specific acupoint ST36 regulates inflammation and its underlying mechanisms. We searched literatures on PubMed until July 2021 using the keywords “animal, acupuncture, ST36, inflammation, immune,” and 292 literatures were searched. We ultimately selected 69 studies to determine the anti-inflammatory actions of acupuncture at ST36 and classified the changes of inflammatory mediators according to target regions. Forty-three studies were included in body fluids, 27 studies in the digestive system, 17 studies in the nervous system, and 30 studies in other tissues or organs. In this review, we found that acupuncture at ST36 has clinical benefits in relieving inflammation through several mechanisms such as vagus nerve activation, toll-like receptor 4 (TLR4)/NF-κB signaling, macrophage polarization, mitogen-activated protein kinase (MAPK) signaling pathway, and cholinergic anti-inflammatory pathway. We expect that these data will inform further studies related to ST36 acupuncture on inflammation.

## Introduction

Inflammation is a physiological protective process that prevents foreign injuries or infections. Inflammation includes not only an inflammatory response but also the step of restoring tissues. The biological progression of inflammation is composed of diverse inflammatory cytokines and chemokines. Tumor necrosis factor-α (TNF-α) is produced by inflammatory cells and induces encoding genes of antiapoptotic molecules. IL-6 is one of the inflammatory cytokines, and STAT3 plays a critical role in its signal transduction (1). Immune cells are deeply involved in the inflammation triggered by external or endogenous stimuli. Macrophages play a critical role in the maintenance of tissue homeostasis and are composed of two subsets: M1 macrophages, which produce proinflammatory cytokines, and M2 macrophages, which promote tissue repairs and secrete inflammation-suppressive mediators (2). T cells are separated by their actions into cytotoxic T cells, which engage in defense against intracellular pathogens, and helper T cells, which assist macrophage activations (3). Since prolonged chronic inflammation can lead to irreparable damage to tissues and aggravate the disease status, operation of the rapid immune system and normal regulation of inflammatory mediators is important (4).

Typically, non-steroidal anti-inflammatory drugs (NSAIDs) are widely used in the modern period to combat inflammation (5). NSAIDs are therapeutic medicines synthesized to competitively inhibit cyclooxygenases (COXs), which results in blocking the synthesis of proinflammatory prostaglandins (6). However, the use of NSAIDs is closer to relieving pathological symptoms rather than a complete cure of diseases (7). Furthermore, some researchers have studied that NSAIDs have adverse effects on gastrointestinal tracts (8) or respiratory organs (9) and are linked to hepatotoxicity (10). The long-term usage of NSAIDs is reported to commonly cause gastric hemorrhage (11) and increase the risk of kidney injuries (12). Therefore, acupuncture therapy is becoming gradually popular as one of the alternative therapies for treating diseases with fewer side effects.

In traditional Chinese medicine (TCM), acupuncture is one of important therapeutic techniques. Acupuncture practice involves the action of inserting needles into specific points, called “acupoints”, and subsequently electrical stimulation or manual techniques can be applied. In the aspect of medical application, acupuncture therapy has been used to alleviate pain and treat various diseases such as rheumatoid arthritis (RA) (13), allergic rhinitis (14), atopic dermatitis (15), chronic pain (16), diabetic neuropathy (17), and other infectious diseases (18). ST36 (Zusanli in Chinese) is one of the well-known acupoints, which is located on the anterior aspect of the leg, on the line connecting ST35 with ST41, 3 B-cun inferior to ST35, and on the tibialis anterior muscle (19). The numerous efficacies of acupuncture at ST36 like anti-inflammation effect (20), anti-oxidation effect (21), enhancing immune system (22), and promoting the restoration of postoperative gastrointestinal functions (23) have been reported. Especially, the latest studies revealed that electroacupuncture (EA) at ST36 modulates endotoxin-induced systemic inflammation *via* driving the distinct sympathetic pathways and making the mechanism of the vagal–adrenal anti-inflammatory axis known (24, 25).

There is a recent study that systematically reviewed acupuncture at ST36 as a treatment for sepsis. This systemic review is just confined to sepsis models (26), so it is hard to know the effects of acupuncture on overall inflammation. Since there is no literature review that organized the effects of acupuncture at ST36 on the whole inflammatory models, we totally investigated the inflammation-related biomarkers in target regions and suggested that acupuncture at ST36 could be a clinical treatment of inflammatory disorders. Therefore, we investigated animal model studies associated with the anti-inflammatory effects of acupuncture at ST36 and analyzed how acupuncture ST36 has anti-inflammatory effects and its underlying mechanisms.

## Material and Methods

### Eligibility Criteria

All qualified studies included the keywords animal, ST36, acupuncture, inflammation, and immune. Two authors checked the quality of the studies in relation to the methodology, statistics, and the display of the results. Studies that used animal models except rodent models were excluded from this study.

### Study Selection

**Figure 1:**
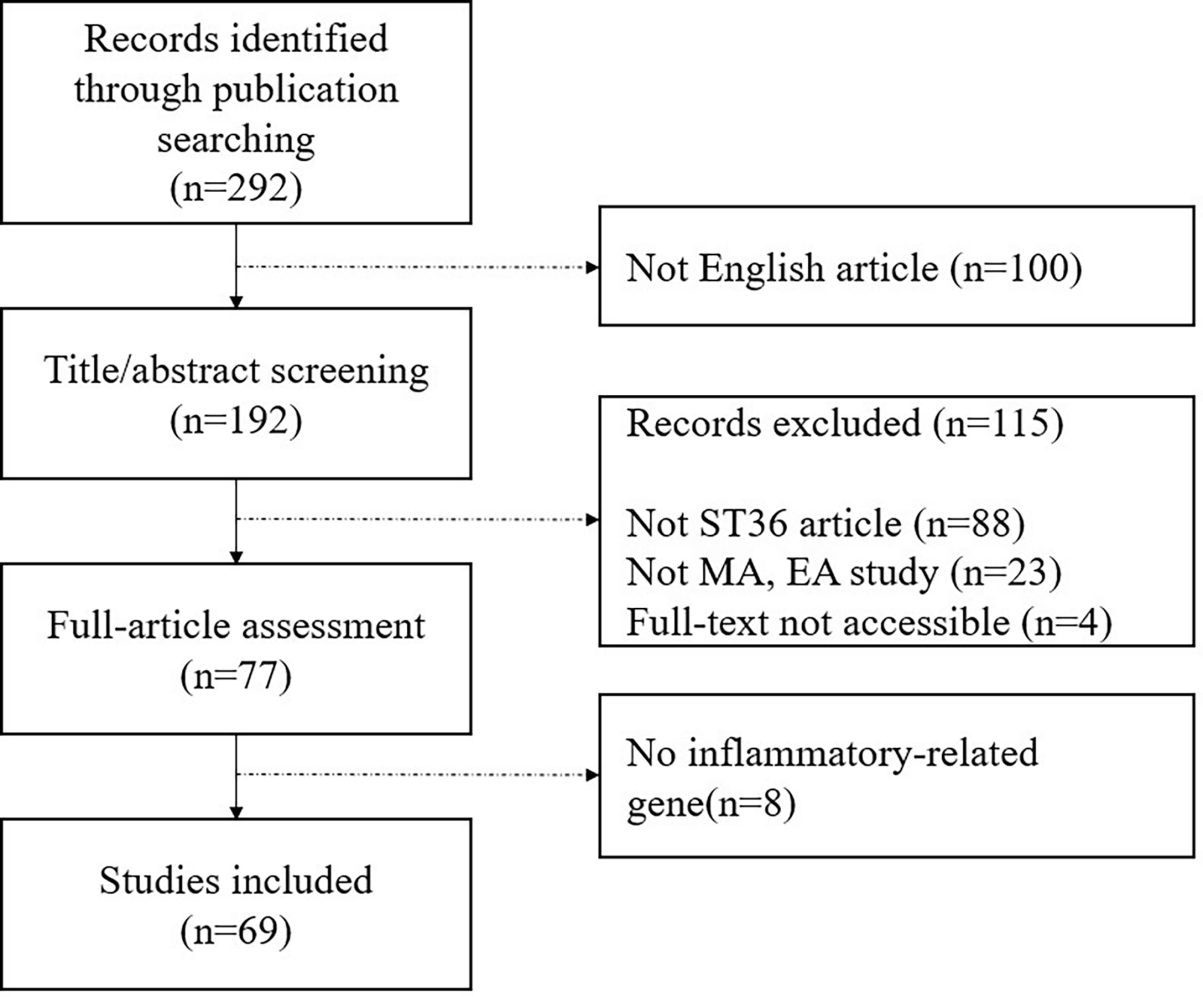
Flowchart of study selection.

We identified literatures on EMBASE, MEDLINE, and PUBMED since inception using the search terms “(acupuncture OR electroacupuncture) AND (mouse OR rat OR mice OR rats) AND (inflammation OR inflammatory OR immune OR immunological OR immunology) AND (ST36 OR Zusanli).” Two independent reviewers conducted the research independently for this study. **Figure 1** shows a flowchart of study selection for this review. We found 292 potentially relevant literatures *via* searching on an online database and of these articles, we excluded 223 articles for the following reasons: 1) not English articles; 2) articles that did not use ST36 only; 3) studies that did not use manual acupuncture (MA) and EA; 4) full text was not accessible; and 5) not inflammatory-related gene studies. At last, 69 articles were included in this study.

## Results

### Body Fluids

One of the mechanisms that explain how acupuncture produces anti-inflammatory effects is that acupuncture makes local inflammatory reactions in the acupoint, amplifies the signal transmission, acts on the whole body, and ultimately makes acupuncture effects. In order to oversee how acupuncture regulates systemic inflammation, we investigated the change of inflammatory mediators in body fluids. Among the total 69 articles included in this analysis, 43 articles observed how acupuncture at ST36 changed inflammation-related substances in body fluids (**Table 1**).

**Table 1:** Anti-inflammatory effect of acupuncture on ST36 in body fluids.

Author	Year	Mouse Model	Acupuncture Method	Target	Inflammatory Markers
Zhang Z, et al. (22)	2021	BALB/c mice with breast tumors	2/15-Hz electroacupuncture (EA)	Serum	IL-1β, TNF-α levels ▽ IL-10 levels △
				Blood	Proportions of CD8+ T cells (CD3e+CD8a+) △ Proportions of NK cells (CD3e−CD49b+) △ Proportions of MDSCs* (CD11b+Gr-1+) ▽
Zhang L, et al. (27)	2021	Caerulein-induced acute pancreatitis (AP)	2/15-Hz EA	Plasma	LF/HF* of HRV* ▽ Amylase level ▽ TNF-α, IL-1β, IL-6 levels ▽
		Pancreatic duct ligation (PDL)-induced pancreatitis	2/15-Hz EA	Plasma	Amylase level ▽ TNF-α, IL-1β, IL-6 levels ▽
Li Y, et al. (28)	2021	Ischemia–reperfusion (gut ischemia)	10-Hz EA	Plasma	Dopamine concentration △ TNF-α concentration ▽ Plasma dextran (intestinal permeability to 4-kDa FITC* dextran) ▽
Xie DP, et al. (29)	2020	Cecal ligation and puncture (CLP)	3-Hz EA	Serum	TNF-α concentration ▽ IL-10 concentration ▽ D-LA* concentration ▽ DAO* concentration ▽
				Mesenteric lymph nodes	CD3+CD4+ cells/CD3+CD8+ cells ratio △ Treg/Th17 cells ratio △
Liu GH, et al. (30)	2020	Dextran sulfate sodium (DSS)-induced colitis	2-Hz EA	Plasma	CRP* (inflammatory biomarker) ▽ IFN-γ, TNF-α, IL-6 (proinflammatory cytokines) ▽ adiponectin △
Lim HD, et al. (31)	2020	Concanavalin A (ConA) model of hepatitis	Manual acupuncture (MA) 1-Hz, 5-Hz EA	Serum	TNF-α* ▽
Xie LL, et al. (32)	2020	Diet-induced obese knee osteoarthritis models (DIO-KOA)	30-Hz EA	Serum	TC* ▽ TG* ▽ HDL* △ VEGF, MIP-1α, MIP-2, IP-10, IL-1β, TNF-α, MCP-1*, leptin ▽
				Synovial fluid	VEGF, MIP-1α, MIP-2, IP-10, IL-1β, TNF-α, MCP-1, leptin ▽
Wang L, et al. (33)	2020	DSS-induced chronic colitis	10-Hz EA 100-Hz EA	Serum	IL-10 level △ IL-6 level ▽
				Plasma	FD4 level ▽
Yang NN, et al. (34)	2020	Postoperative ileus (POI) model	2-Hz, 10-Hz, 30-Hz, 100-Hz EA	Serum	TNF-α concentration ▽ IL-6 concentration ▽
Zhao YX, et al. (35)	2020	Pentoxifylline (PTX)-injected mechanical allodynia (PTX-induced neuropathic pain)	10-Hz EA	Serum	IL-1β ▽ TNF-α ▽
Harpin D, et al. (36)	2020	Wistar sepsis model	2-Hz EA	Blood	Urea ▽ Creatinine ▽
Jin H, et al. (37)	2019	Intrarectal administration of trinitrobenzenesulfonic acid (TNBS)	25-Hz EA 5-Hz EA	Plasma	TNF-α level ▽ IL-6 level ▽ IL-1β level ▽
Song S, et al. (38)	2019	DSS-induced acute colitis	10-Hz EA 100-Hz EA	Serum	IL-1β ▽ TNF-α ▽ IL-6 ▽ IL-12 ▽
Wang Z, et al. (39)	2019	2,4-Dinitrofluorobenzene (DNFB)-induced allergic contact dermatitis (ACD)	2-Hz EA	Serum	IgE level ▽
Chen T, et al. (40)	2019	Lipopolysaccharide (LPS)-induced inflammation	2-Hz EA	Serum	TNF-α, IL-1β, IL-6 ▽ Ca^2+^ ▽ TLR4, NF-κB p65 ▽
Zhang K, et al. (41)	2018	Complete Freund’s adjuvant (CFA)-induced inflammation	MA	Serum	NPY ▽ MCP-1 △ GRO/KC △ PRL ▽
Tian L, et al. (42)	2018	Streptozotocin (STZ)-injected type 1 diabetic mellitus	10-Hz EA 100-Hz EA	Serum	MDA* concentration ▽
Zhang L, et al. (43)	2018	Myocardial injury sepsis model	2/100-Hz EA	Plasma	CK-MB* ▽
Chen L, et al. (44)	2017	Sprague–Dawley (SD) rats	2-Hz, 15-Hz EA	Serum	IFN-γ, IL-2, IL-17 △ Ca^2+^ ▽
Geng Y, et al. (45)	2017	Intestinal ischemia–reperfusion (I/R) injury	2/100-Hz EA	Serum	IL-6 ▽ TNF-α ▽
Wang Z, et al. (46)	2017	DNFB-induced ACD	2-Hz EA	Serum	IgE level ▽
Wang Z, et al. (47)	2017	Ovalbumin (OVA)-induced delayed-type hypersensitivity (DTH)	2-Hz EA	Serum	IgE ▽ OVA-specific IgG ▽
Lim HD, et al. (48)	2016	LPS administration endotoxemia	MA 1-Hz EA	Serum	TNF-α ▽
Liu M, et al. (49)	2016	Chronic psychological stress (CPS) model	2/15-Hz EA	Serum	IgG, IgM, IL-2 △ IL-6 ▽
Geng Y, et al. (50)	2016	Intestinal I/R injury	2/100-Hz EA	Serum	IL-6 ▽ TNF-α ▽
Wen CK, et al. (51)	2015	Obese leptin-deficient mice	2-Hz EA	Serum	Insulin, glucose, triglyceride, total cholesterol ▽ TNF-α, IL-6, IL-1β ▽
Zhu MF, et al. (52)	2015	CLP-induced sepsis model	2/100-Hz EA	Serum	d-Lactose concentration ▽
Hu S, et al. (53)	2015	Hemorrhagic shock (HS)	2/100-Hz EA	Plasma	Plasma dextran (intestinal permeability to 4-kDa FITC dextran) ▽
Song XM, et al. (54)	2015	Total body surface area (TBSA) scald subjected thermal injury (TEM)	3-Hz EA	Plasma	IL-1β ▽ IL-6 ▽ HMGB-1 ▽
Villegas-Bastida A, et al. (55)	2014	CLP-induced polymicrobial sepsis model	30-Hz EA	Serum	TNF level ▽ IL-6 level ▽ Nitrite level ▽ HMGB-1 level ▽
Wen CK, et al. (56)	2014	High-fat diet (HFD)-induced obesity model	10-Hz EA	Plasma	ALT*, AST* ▽ Total cholesterol, triglyceride, free fatty acid ▽ TNF-α, IL-1, IL-6 ▽
Peng MF, et al. (57)	2014	Laparotomy-operated rats	5/20-Hz EA	Serum	TNF-α activity ▽ NO activity ▽
Xue QM, et al. (58)	2014	Severe acute pancreatitis (SAP) model	2/100-Hz EA	Serum	TNF-α ▽ IL-6 ▽ Ach △
Song Q, et al. (59)	2014	LPS endotoxin challenge	2/100-Hz EA	Plasma	TNF-α ▽
				Serum	ALT, CK-MB, Cr, DAO (heart, liver, kidney, intestine function parameters) ▽
Du MH, et al. (60)	2013	HS	2/100-Hz EA	Plasma	TNF-α, IL-6 ▽ IL-10 △ Plasma dextran (permeability to 4-kDa FITC dextran) ▽ ALT* ▽ CK-MB* ▽ Cr* ▽
Hu S, et al. (61)	2013	Intestinal I/R model	2/100-Hz EA	Plasma	TNF-α ▽ IL-8 ▽ Plasma dextran (intestinal permeability to 4-kDa FITC dextran) ▽
Geng WY, et al. (62)	2013	Chronic obstructive pulmonary disease (COPD)	2/60-Hz EA	Bronchoalveolar lavage fluid	TNF-α ▽ IL-1β ▽ MDA* (lipid oxidation product) ▽
Gim GT, et al. (63)	2011	Neuropathic surgery	2-Hz EA	Serum	IgG concentration △
Liu YM, et al. (64)	2010	Experimental autoimmune encephalitis	1-Hz EA	Supernatant	IFN-γ, IL-17 ▽ IL-4, TGF-β △
				Lymphocyte	T-cell proliferation ▽ Th1 (CD4+IFN-γ+ T cell), Th17 (CD4+IL-17+ T cell) ▽ Treg (CD4+Foxp3+ T cell), Th2 (CD4+IL-4+ T cell) △
				Plasma	ACTH △
An HJ, et al. (65)	2007	Cholecystokinin (CCK)-induced acute pancreatitis	100-Hz EA 2-Hz EA	Serum	β-Amylase, lipase ▽ ACTH release △
Chae Y, et al. (66)	2007	Carrageenan-induced paw edema	MA	Serum	IL-6 protein level ▽ NGF* protein level ▽ TIMP*-1 protein level ▽
Yim YK, et al. (67)	2007	Collagen-induced arthritis (CIA)	2-Hz EA	Serum	IL-6, IgG, IFN-γ, IgM, TNF-α, Collagen 2 Antibody ▽
				Lymph node	CD69+/CD3e+ cell populations ▽ CD11a+/CD19+ cell populations ▽ CD3e+/CD19+ ratio △ CD4+/CD8+ ratio △
Tian L, et al (68).	2003	TNBS-induced ulcerative colitis (UC)	2-Hz EA	Serum	TNF-α concentration ▽

*MDSCs, myeloid-derived suppressor cells; LF/HF, low frequency/high frequency; HRV, heart rate variability; FITC, fluorescein isothiocyanate; D-LA, D-lactic acidosis; DAO, double amine oxidase; CRP, C-reactive protein; TNF-a, tumor necrosis factor-a; TC, total cholesterol; TG, triglyceride; HDL, high-density lipoprotein; MCP-1, monocyte chemotactic protein 1; MDA, malondialdehyde; CK-MB, creatine kinase-MB; ALT, alanine transaminase; AST, aspartate transaminase; Cr, creatinine; NGF, nerve growth factor; TIMP, tissue inhibitors of metalloproteinase.

In the cecal ligation and puncture (CLP) model, EA at ST36 reduced both serum levels of TNF-α and IL-10, which abnormally increased by systemic inflammation. Also, EA reduced d-lactic acidosis (D-LA) and double amine oxidase (DAO) concentration in serum, which means EA may strengthen the intestinal barrier. Since the pre-splenectomy was conducted, the anti-inflammatory effect of EA disappeared; this study showed that in the mechanism of EA at ST36, the spleen plays an important role in regulating systemic inflammation (29). In another CLP-induced polymicrobial sepsis model, EA downregulated the level of TNF-α, IL-6, nitrite, and high-mobility group box 1 (HMGB-1) in serum, and reduced nuclear fraction NF-κB p65 activity in the spleen. However, when the ectomy of the subdiaphragmatic vagus nerve and the injection of reserpine, which pharmacologically inhibits catecholamine production, were performed together, the TNF level of serum did not show a significant reduction as compared with the EA group. This showed that the effect of acupuncture treatment at ST36 depends on vagus nerve integrity and the production of catecholamines (55).

In the dextran sulfate sodium (DSS)-induced colitis model, EA suppressed proinflammatory cytokines including IFN-γ, TNF-α, and IL-6. EA also reduced the increased level of C-reactive protein (CRP), which is stimulated when inflammatory diseases occurred. This study showed that EA at ST36 has an anti-inflammatory effect on the toll-like receptor 4 (TLR4)/NF-κB signaling pathway (30).

In the diet-induced obese knee osteoarthritis (DIO-KOA) model, ST36 EA reduced total cholesterol and triglyceride level in serum but increased high-density lipoprotein (HDL) levels in serum. Subsequently, EA decreased vascular endothelial growth factor (VEGF) and catabolic enzymes MIP-1α and MIP-2, which promote joint inflammation and reduced key proinflammatory cytokines such as IP-10, IL-1β, TNF-α, leptin, and monocyte chemokine MCP-1 in both serum and synovial fluid. Also, EA decreased matrix metalloproteinase (MMP)-1, MMP-13, TLR-4, NF-κB p65, and NF-κB P-p65 expressions in the arthrodial cartilage of the knee joint. MMP-1 and MMP-13 are catabolic enzymes, have cartilage-destructive properties, and are secreted by VEGF release. The above findings indicate that acupuncture can attenuate synovial inflammation by regulating lipid metabolism and joint inflammation *via* suppressing TLR4/NF-κB signaling pathway (32).

In the ischemia–reperfusion (I/R) model, EA at ST36 increased the level of dopamine and decreased TNF-α concentration in plasma. Also, EA decreased the activity of myeloperoxidase (MPO) and malondialdehyde (MDA) in the intestines, indicating intestinal protective effects. In this study, when vagotomy (VGX) or butaclamol injection, which is a D1 receptor antagonist, was conducted, the EA anti-inflammatory effect was diminished or weakened. This suggested that the EA effect is mediated through activation of the vagus nerve and D1 receptors, and EA regulates the adrenal medulla leading to the release of dopamine and inhibiting cytokine production (28). In another intestine-ischemia model, EA lowered TNF-α, IL-8, intestinal permeability to fluorescein isothiocyanate (FITC) dextran in plasma and increased intestinal ZO-1 protein expression. Also, EA decreased TNF-α and IL-8 in both lung and liver, but these effects were reversed when abdominal VGX and intraperitoneal administration of cholinergic α7 nicotinic acetylcholine receptor (α7nAChR) inhibitor were conducted. This finding indicates that EA alleviates systemic inflammation *via* the intact vagus nerve and α7nAChR (61).

In the Wistar sepsis model, EA reduced the levels of urea and creatinine in the blood, which are parameters of kidney function, and it showed that EA may prevent kidney injuries (36).

In the lipopolysaccharide (LPS)-induced inflammation model, ST36 EA decreased the level of proinflammatory cytokines such as TNF-α, IL-6, and IL-1β and decreased Ca^2+^, TLR4, and NF-κB p65 expression in serum. In spleen mononuclear cells, EA reduced Ca^2+^ influx and TLR4 expression and increased CB2R expression. In this study, the mechanism of acupuncture at ST36 suggests that EA inactivates TLR4/NF-κB signaling pathway by increasing the expression of CB2 receptors and inhibiting Ca^2+^ influx (40).

This study used the complete Freund’s adjuvant (CFA)-induced inflammation model and revealed how MA at ST36 modulates neuroendocrine–immune (NEI) networks in relation to main signaling molecules. MA decreased the level of neuropeptide Y (NPY), monocyte chemotactic protein 1 (MCP-1), growth-related oncogene/keratinocyte-derived chemokines (GRO/KC), and prolactin (PRL) in serum. Also, MA decreased brain-derived neurotrophic factor (BDNF), regulated on activation normal T cell expressed and secreted (RANTES), macrophage colony-stimulating factor (M-CSF), and increased corticotropin-releasing hormone (CRH) in hind footpad tissues. As a physical stimulation, MA induced the increment of hormones such as thyroid-stimulating hormone (TSH), corticosterone, follicle-stimulating hormone (FSH), melatonin, and proinflammatory cytokines including IL-1β and IL-6, GRO/KC, and M-CSF in ST36 tissues. These changes of key molecules at local acupoint triggered signaling cascades acting on the NEI network and eventually showed analgesic and anti-inflammatory effects of acupuncture (41).

In the chronic psychological stress (CPS) model, EA at ST36 increased IgG, IgM, and IL-2 but decreased IL-6 in serum. Also, EA increased the number of interstitial cells of Cajal (ICCs), synapses of ICCs, and integrated optical density (IOD) of ICCs in intestine tissues. ICCs play a crucial role in modulating gastrointestinal motilities, and these findings show that EA is able to repair ICC damages. In this study, the regulation effect on immune responses of EA at ST36 is related to visceral hypersensitivity. EA regulated the immunity by improving visceral hypersensitivity *via* signaling cascades in the neuroendocrine system (49).

In the total body surface area (TBSA) scald subjected thermal injury (TEM) model, EA downregulated the level of IL-1β, IL-6, and HMGB-1 in plasma. Also, EA lowered HMGB-1 protein and mRNA expression and inflammatory cellular infiltration in lung tissues including bronchial epithelium and luminal surface. However, in the α7nAChR subunit antagonist (α-BGT)-administered group, the changes of proinflammatory cytokines in plasma were not found in this study. The data in this experiment showed that EA at ST36 limited the protein expression of HMGB-1, which is the main endotoxin mediator and suppressed inflammatory responses *via* cholinergic anti-inflammatory pathway related with α7nAChR subunit (54).

In laparotomy-operated rats, EA at ST36 significantly decreased TNF-α and nitric oxide (NO) activity in serum and increased slow-wave myoelectric activity and c-Kit protein expression in intestinal segments. This study suggested that EA at ST36 expressed tyrosine kinase receptor c-Kit protein and activated ICC cellular networks to function through resident macrophages. The increase of ICC numbers led to the decline of inflammatory mediator TNF-α. That is, the EA effect was mediated *via* the c-Kit signaling pathway (57).

In the severe acute pancreatitis (SAP) model, EA decreased the level of TNF-α and IL-6 in serum but increased acetylcholine (Ach) levels in serum. Also, EA showed moderate necrosis, hemorrhage, and leukocyte infiltrations in the pancreatic structure. Ach, as an important vagus nerve neurotransmitter, is known to suppress the secretion of proinflammatory cytokines. This research demonstrated that EA at ST36 could attenuate inflammatory responses through the cholinergic anti-inflammatory pathway (58).

In the LPS endotoxin challenge model, EA at ST36 reduced TNF-α in plasma and increased alanine aminotransferase (ALT), creatine kinase-MB (CK-MB), Cr, and DAO levels in serum, which are the parameters of heart, liver, kidney, and intestine function, respectively. However, the anti-inflammatory effect and organ-protective effect of EA at ST36 were aggravated when the α7 subunit of cholinergic N receptor was incapacitated by the antagonist α-BGT, and VGX was performed. The data in this study concluded that ST36 EA is able to protect organ dysfunction and show anti-inflammatory effects mainly through the cholinergic anti-inflammatory pathway by releasing Ach (59).

In the hemorrhagic shock (HS) mouse model, EA at ST36 reduced TNF-α, IL-6, ALT, CK-MB, and Cr in plasma; TNF-α and IL-6 in the intestine; and plasma dextran, which means intestinal permeability to 4 kDa of FITC dextran. EA increased ZO-1 protein expression in the intestine and IL-10 in both plasma and the intestine. However, when the treatment of abdominal VGX and α-BGT injection was performed, EA effects were weakened or eliminated. These data indicated that ST36 EA could attenuate systemic inflammation and improve organ functions through the cholinergic anti-inflammatory pathway especially *via* the vagus nerve and α7nAChR (60).

In the COPD model, ST36 EA downregulated the levels of TNF-α, IL-1β, and MDA in bronchoalveolar lavage fluid (BALF). The reduction of MDA level, which is a product of lipid oxidation, indicates that EA at ST36 shows an antioxidant effect. EA also reduced bronchi and bronchiole obstruction of lung histopathological sections and improved lung function with decreased lung resistance and increased lung compliance. This finding suggests that EA at ST36 can improve lung function and modulate inflammatory cytokine levels through antioxidant mechanisms (62).

In the experimental autoimmune encephalitis model, EA at ST36 reduced the cellular infiltrates in spinal cords and increased adrenocorticotropic hormone (ACTH) level in both the hypothalamus and plasma. EA also regulated the concentration of inflammation-related cytokines in supernatants, reducing IFN-γ and IL-17 and increasing IL-4 and TGF-β. Moreover, EA regulated T-cell proliferation, decreasing CD4+IFN-γ+ T cell representing Th1 cells and CD4+IL-18+ T cell representing Th17 cells and increasing CD4+Foxp3+ T cell representing Treg cells and CD4+IL-4+ T cell representing Th2 cells. This study showed that EA stimulation can restore the balance of Th1/Th2/Th17/Treg/Th cells through modulating the hypothalamus and increasing the secretion of ACTH. Also, this study suggests that the β-endorphin secretions by EA stimulation are relevant to modulating inflammatory responses (64).

In the cholecystokinin (CCK)-induced acute pancreatitis model, EA at ST36 reduced β-amylase and lipase levels in serum, which significantly rise with the symptoms of acute pancreatitis. EA showed these clinical effects against pancreatitis through increasing ACTH release in serum. Also, EA upregulated HSP60 and HSP72 expression and downregulated TNF-α and IL-1β in the pancreas, which demonstrates the protective effect for the pancreas. These results suggest that EA stimulates the hypothalamus or dorsal vagal networks and leads to the release of melanocortin ACTH, resulting in suppression of NF-κB signaling transcription and proinflammatory cytokine production (65).

In the carrageenan-induced paw edema model, ST36 MA decreased IL-6, β-nerve growth factor (β-NGF), and tissue inhibitors of metalloproteinase (TIMP)-1 protein level in serum. NGF triggers the proliferation of T and B cells and is involved in the differentiation of peripheral afferent neurons and sympathetic neurons; in addition, TIMP-1 is induced by proinflammatory cytokines. This study indicated that the anti-inflammatory actions of MA at ST36 are associated with regulating inflammation-relevant cytokine expressions (66).

In the collagen II immunization-induced arthritis (CIA) model, EA at ST36 reduced IL-6, IgG, IFN-γ, IgM, TNF-α, and collagen II antibody in serum and showed histologically relieved milder changes in knee joint. In addition, EA reduced CD69+/CD3e+ cell populations and CD11a+/CD19+ cell populations in lymph nodes, indicating the decline of activated T and B cells that induce the secretion of proinflammatory cytokines, whereas EA increased CD3e+/CD10+ ratio and CD4+/CD8+ ratio in a mouse lymph node, indicating the recovery of T cells/B cells ratio close to the normal range. These findings demonstrate that EA modulates immune abnormalities and potentially treats autoimmune diseases (67).

In trinitrobenzenesulfonic acid (TNBS)-induced ulcerative colitis (UC) model, ST36 EA decreased the concentration of TNF-α in serum and MPO activities, and TNF-α mRNA expression in the colon. The elevated index of inflammation and MPO activity was reduced by downregulating anti-inflammatory cytokines and inhibiting systemic inflammation cascades. The therapeutic effect of EA is attributed to the modulation of the balance between anti-inflammatory cytokines and proinflammatory cytokines (68).

### Digestive System

Of the total 69 studies, 27 studies are involved in the anti-inflammatory effect on the digestive system of acupuncture on ST36 (**Table 2**).

**Table 2:** Anti-inflammatory effect of acupuncture on ST36 in digestive system.

Author	Year	Mouse Model	Acupuncture Method	Target	Inflammatory Markers
Zhang Z, et al. (22)	2021	BALB/c mice with breast tumors	2/15-Hz electroacupuncture (EA)	Spleen	Proportions of CD8+ T cells (CD3e+CD8a+) △ Proportions of NK cells (CD3e−CD49b+) △ Proportions of MDSCs (CD11b+Gr-1+) ▽
Zhang L, et al. (27)	2021	Caerulein-induced acute pancreatitis (AP)	2/15-Hz EA	Pancreas	LF/HF of HRV ▽ Histology score (edema, inflammation, acinar necrosis, hemorrhage) ▽ Percentage of CD11b+Ly6G+ neutrophils, percentage of CD11b+F4/80+ macrophages ▽ Percentage of α7nAchR+ macrophages △
		Pancreatic duct ligation (PDL)-induced pancreatitis	2/15-Hz EA	Pancreas	Histology score (edema, inflammation, acinar necrosis, hemorrhage) ▽
Chen Y, et al. (69)	2021	Dextran sulfate sodium (DSS)-induced intestinal inflammation	100-Hz, 25-Hz, 5-Hz EA	Distal colon	Tryptase+ cell expression (tryptase-positive area) ▽ Activated mast cell expression (mast cell activation rate) ▽ TrkA+ expression area (TrkA-positive area) ▽ TrkA protein expression ▽ NGF protein expression ▽ TRPV1+PGP9.5+ neurons expression (TRPV1/PGP.5-positive area) ▽
Li Y, et al. (28)	2021	Ischemia–reperfusion (gut ischemia)	10-Hz EA	Intestine	MPO* ▽ MDA ▽
Wang L, et al. (33)	2020	DSS-induced chronic colitis	10-Hz EA 100-Hz EA	Colon	IL-10 protein expression △ TNF-α protein, mRNA expression ▽ IL-1β protein, mRNA expression ▽ iNOS protein, mRNA expression ▽ IL-6 mRNA expression ▽ ZO-1, Occludin, E-cadherin protein expression △ ZO-1, Occludin, MUC2 protein, mRNA expression △ The number of TUNEL-positive and apoptotic cells ▽ Ki67-positive IECs (intestinal epithelial cells) proliferation ▽ P-ERK1, 2, P-JNK, P-p38, P-STAT3 protein expression △
Yang NN, et al. (34)	2020	Postoperative ileus (POI) model	2-Hz, 10-Hz, 30-Hz, 100-Hz EA	Intestine	MPO activity ▽
Jin H, et al. (37)	2019	Intrarectal administration of trinitrobenzenesulfonic acid (TNBS)	25-Hz EA 5-Hz EA	Colon tissue	MPO activity ▽
Song S, et al. (38)	2019	DSS-induced acute colitis	10-Hz EA 100-Hz EA	Colon	IL-1β, TNF-α, IL-17 protein ▽ IL-12 protein △ IL-1β, TNF-α, IL-6, IL-12, IL-17, iNOS (M1-associated genes) mRNA ▽ IL-10 mRNA △
				Colonic lamina propria mononuclear cells (LPMCs)	CD206, Arg-1, FIZZ1 (M2-associated genes) in macrophages △ CD16/32+ (M1 macrophage) activation ▽
				Colonic tissue macrophages	CD206+CD16/32− (M2 macrophage) activation △ NLRP3 protein, cleaved IL-1B, cleaved caspase1 ▽ NLRP3 mRNA, IL-1β mRNA ▽ MOD of NLRP3 (NLRP3 activation) ▽ MOD of HO-1 (HO-1 secretion) △
Wang Z, et al. (39)	2019	2,4-Dinitrofluorobenzene (DNFB)-induced allergic contact dermatitis (ACD)	2-Hz EA	Rat peritoneal mast cells (RPMCs)	CB2R mRNA, protein △ Mast cell infiltration ▽ β-Hexosaminidase, histamine release ▽ p-p38 protein expression ▽
Wang Z, et al. (70)	2018	DNFB-induced ACD	2-Hz EA	IL-33-obtained RPMCs	IL-6, TNF-α, IL-13, MCP-1 ▽
				Cytosol of peritoneal mast cells (RPMCs)	IkBa expression △ p-IKKa/B, p-P38 expression ▽
				Nucleus of peritoneal mast cells (RPMCs)	NF-κB p65, p-c-Jun expression ▽ miR-155 expression ▽
Tian L, et al. (42)	2018	Streptozotocin (STZ)-injected type 1 diabetic mellitus	10-Hz EA 100-Hz EA	Stomach tissue	Mean density of ICC-MY △ HO-1 protein expression △ HO-1 relative mRNA expression △ F4/80 (HO-1-positive macrophages) △ IL-10 protein △ IL-10 relative mRNA expression △ CD163 mRNA expression △ Arg-1* mRNA expression △ iNOS mRNA expression ▽
Geng Y, et al. (45)	2017	Intestinal ischemia–reperfusion (I/R) injury	2/100-Hz EA	Small intestine	α7nAChR* mRNA expression △ NF-κB p65 ▽
Geng Y, et al. (50)	2016	Intestinal I/R injury	2/100-Hz EA	Intestinal crypt cells	Ki67 proliferation index △
				Distal ileum mucosa	SDF-1 mRNA expression △ CXCR4 mRNA expression △ EGF mRNA expression △ EGFR mRNA expression △ NF-κB p65 mRNA expression ▽
Liu M, et al. (49)	2016	Chronic psychological stress (CPS) model	2/15-Hz EA	Intestine tissue	Number of ICCs* △ Synapses of ICCs △ IOD* of ICCs △
Du MH, et al. (71)	2015	Intra-abdominal adhesion formation	2/100-Hz EA	Cecal adhesive tissue	TNF-α ▽ VEGF ▽ CD31+ ▽ Microvessel count ▽
Hu S, et al. (53)	2015	Hemorrhagic shock (HS)	2/100-Hz EA	Intestine (intestinal extracts)	GFAP* expression △ GFAP mRNA expression △ TNF-α level ▽ ZO*-1 protein expression △
Zhu MF, et al. (52)	2015	CLP-induced sepsis model	2/100-Hz EA	Intestinal mucosa cells	sIgA level △ Percentage of CD3+ T lymphocytes △ Percentage of r/d T lymphocytes △ Percentage of CD4+ T lymphocytes △ Percentage of CD8+ T lymphocytes △ Ratio of CD4+/CD8+ T cells △
Goes AC, et al. (72)	2014	Colitis induced by TNBS	100-Hz EA	Intestine tissue (colon tissue)	MPO activity ▽ IL-1β concentration ▽ IL-10 concentration △ iNOS expression ▽ MDA concentration ▽
Peng MF, et al. (57)	2014	Laparotomy-operated rats	5/20-Hz EA	Intestinal segments	Slow-wave myoelectric activity (amplitude, frequency) △ c-Kit protein expression △
Zhang L, et al. (73)	2014	Postoperative abdominal adhesion formation	2/100-Hz EA	Cecum	TNF-α level ▽ NO level ▽ NOS* activity ▽
Xue QM, et al. (58)	2014	Severe acute pancreatitis (SAP) model	2/100-Hz EA	Pancreas	Necrosis, hemorrhage, leukocyte infiltration ▽
Du MH, et al. (60)	2013	HS	2/100-Hz EA	Intestine	TNF-α ▽ IL-6 ▽ IL-10 △ ZO-1 protein expression △ Intestinal histology ▽
Hu S, et al. (61)	2013	Intestinal I/R model	2/100-Hz EA	Intestine (distal ileum)	TNF-α ▽ IL-8 ▽ Intestinal ZO-1 protein expression △
Aguiar DN, et al. (74)	2012	BALB/c mice with LPS or L. major infection	15/30-Hz EA	Peritoneal cells	Urea production △ Arginase activity △ NO production ▽ Number of parasites-infected macrophages △
				Peritoneal macrophages	IL-4 receptor expression △
Xu X, et al. (75)	2012	Formaldehyde-induced stomachache (visceral pain)	4/16-Hz EA	Pyloric sphincter	NOS absorbance △ AChE absorbance △ VIP absorbance △ CGRP* absorbance ▽
An HJ, et al. (65)	2007	Cholecystokinin (CCK)-induced acute pancreatitis	100-Hz EA 2-Hz EA	Pancreas	HSP60 expression △ HSP72 expression △ TNF-α, IL-1β ▽
Tian L, et al. (68)	2003	TNBS-induced ulcerative colitis (UC)	2-Hz EA	Colon (colonic tissue)	MPO activity ▽ TNF-α mRNA expression ▽

*MPO, myeloperoxidase; Arg-1,arginase-1; α7nAChR, α7 nicotinic acetylcholine receptor; ICCs, interstitial cell of Cajal; IOD, integrated optical density; GFAP, glial fibrillary acidic protein; ZO, zona occludens; NOS, nitric oxide synthase; AChE, acetylcholinesterase; VIP, vasoactive intestinal peptide; CGRP, calcitonin gene-related peptide.

In the caerulein-induced acute pancreatitis (AP) model, low frequency/high frequency (LF/HF) of heart rate variability (HRV) was measured twice (right after EA treatment and 11 h later) as the marker of sympatho-vagal balance. In the AP group, EA significantly decreased LF/HF of plasma and the pancreas, suggesting that EA causes parasympathetic predominance. Also, EA lowered amylase activity of plasma in the AP group at both time points, but it showed slight reductions in VGX-performed AP group. For the beneficial effect of EA on systemic inflammation, the main inflammatory cytokines such as TNF-α, IL-1β, and IL-6 were investigated, and EA remarkably decreased its plasma levels. However, in the VGX-performed AP group, EA could not induce anti-inflammatory effects due to no intact vagus nerve. Eleven hours after the injection of caerulein, the histological manifestation of the pancreas was examined, and it indicated that EA showed a marked reduction of pancreatitis development including edema, leukocyte infiltrations, acinar necrosis, and hemorrhage. Furthermore, EA notably decreased the infiltration of neutrophils (CD11b+Ly6G+) and macrophages (CD11b+F4/80+) and distinctively increased α7nAChR+ macrophages in the pancreas. These effects of EA were abolished in the VGX-performed group. When methyllycaconitine citrate (MLA), which is a selective α7nAChR antagonist, was pretreated, the suppressive effect of EA on plasma amylase, plasma TNF-α, and pancreas histology score was diminished. It shows that EA alleviates pancreatitis *via* α7nAChR. This study also confirmed that EA has protective effects on the pancreatic duct ligation (PDL)-induced pancreatitis model. The decrease of plasma amylase, TNF-α, IL-1β, IL-6, and pancreas histology score was detected after EA treatment, and additionally, EA reduced the infiltration of MPO-positive neutrophils in the lung. This result suggests that EA prevents acute lung injuries, which most commonly occur as a complication of SAP (27).

In the DSS-induced intestinal inflammation model, EA at ST36 decreased TRPV1+ ganglion cell expression in S2–3 dorsal root ganglions (DRGs). Moreover, EA reduced the expression of tryptase+ cells, activated mast cells, TrkA+ protein, which is an NGF receptor, and NGF protein in the distal colon. EA showed these effects by suppressing the activation of mast cells, leading to the decrease of NGF and TrkA proteins. EA also reduced TRPV1+PGP9.5+ neurons in the distal colon, which are expressed in sensory nerve fibers. Through these findings, EA is suggested to be a non-invasive method of rectal hypersensitivity treatment with the mechanism of mast cell-induced NGF/TrkA/TRPV1 peripheral sensory afferent pathway (69). In another DSS-induced chronic colitis model, both LF ST36 EA and HF ST36 EA increased the level of colonic proteins ZO-1, Occludin, E-cadherin, and MUC2, and this finding showed that EA can protect the mucus layer from bacteria penetration. Also, EA reduced the number of TUNEL-positive and apoptotic cells and Ki67-positive intestinal epithelial cell (IEC) proliferation, indicating that EA influences intestinal barrier integrity. Moreover, EA upregulated the protein level of phosphorylation of ERK1/2, JNK, p38, and STAT3 in the colon; downregulated IL-10 and IL-6 levels in serum; downregulated proinflammatory factor expressions including TNF-α, IL-1β, iNOS, and IL-6 in the colon; and upregulated anti-inflammatory mediator IL-10 in the colon. EA treatment exerted these anti-inflammatory effects through promoting ERK1/2/JNK/p38 mitogen-activated protein kinase (MAPK) signaling pathway, MAPK pathway, and STAT3 signaling pathway *via* modulation of the gut microbiota (33). In another study that used DSS-induced acute colitis model, both LF EA (LEA) and HF EA (HEA) decreased the level of IL-1β, TNF-α, IL-6, and IL-12 in serum. EA decreased the expression of IL-1β, TNF-α, IL-17 protein, and mRNA and increased IL-12 protein and IL-10 mRNA, to maintain the balance between proinflammatory cytokines and anti-inflammatory cytokines. Also, EA upregulated CD206, arginase 1 (Arg-1), and FIZZ1 in macrophages, which are M2-associated genes, and CD206+CD16/32 activation, which is considered M2 macrophage, and downregulated CD16/32+ activation considered M1 macrophage, in colonic lamina propria mononuclear cells (LPMCs). This finding showed that EA modulates the polarization of M1 macrophage and M2 macrophage. Furthermore, both LEA and HEA reduced NLRP3 protein, NLRP3 mRNA, cleaved IL-1β, IL-1β mRNA, cleaved caspase1, and MOD of NLRP3 and increased MOD of HO-1 in colonic macrophages; and HEA promoted Nrf2 expression, which is the main transcription factor synthesizing HO-1 to regulate antioxidant responses. Therefore, EA could suppress the activation of the NLRP3 inflammasome, which causes IL-1β production and promotes Nrf2/HO-1 expressions to have anti-inflammatory abilities. The mechanism of EA to attenuate colitis is associated with the inactivation of the NLRP3/IL-1β pathway and improvement of the Nrf2/HO-1 pathway (38).

In the postoperative ileus (POI) model, EA at ST36 decreased TNF-α and IL-6 concentration in serum and MPO activities in the intestine. MPO activity was measured to oversee the infiltration of macrophages and neutrophils, and this result showed that EA suppressed a local immune response. This indicated that ST36 EA effectively relieved systemic inflammation by restraining local intestinal inflammatory responses (34).

In intrarectal administration of TNBS-induced colonic inflammation model, EA at ST36 decreased TNF-α, IL-6, and IL-1β levels in plasma and MPO activities in colonic tissues. Also, EA upregulated vagal activity and downregulated sympathetic activity, indicating the improvement of sympatho-vagal imbalance. It showed that EA inhibited proinflammatory cytokines through activating autonomic mechanisms like vagal nerve stimulation (37).

In 2,4-dinitrofluorobenzene (DNFB)-induced allergic contact dermatitis (ACD) model, EA at ST36 decreased IgE level in serum and increased CB2R mRNA and protein expression in rat peritoneal mast cells (RPMCs). EA decreased the mast cell infiltration, the release of β-hexosaminidase and histamine, and p-p38 protein expression in RPMCs and also decreased the number of mast cells in ear tissues and ear swelling. However, AM630, a CB2R antagonist, abrogated EA effects inhibiting mast cell degranulation and infiltration, leading to the increase of the release of β-hexosaminidase and histamine. This result suggests that CB2R activation participates in suppressing mast cell degranulation. EA may treat inflammatory skin diseases by enhancing CB2R expression followed by inhibiting the p38 MAPK pathway (39). Another study that used the DNFB-induced ACD model and IL-33 treatment in RPMCs revealed a crucial role of miR-155 in the anti-inflammatory effects of EA. ST36 EA lowered the number of mast cells and mast cell infiltration and the level of IL-33 in ear tissues. EA significantly downregulated IL-33-induced inflammatory cytokines such as IL-6, TNF-α, IL-13, and MCP-1 in RPMCs. EA increased cytosolic IκBα expression and decreased cytosolic p-lKKα/β, cytosolic p-P38 expression, nuclear NF-κB p65, nuclear p-c-Jun expression, and nuclear miR-155 expression. This study demonstrated the mechanism of the anti-inflammatory effect of EA in restraining NF-κB and AP-1 (c-Jun) activation through suppressing miR-155 expression in mast cells (70).

In streptozotocin (STZ)-injected type 1 diabetic mellitus model, EA at ST36 reduced MDA concentration in serum. Also, EA decreased the mean density of ICC-MY, HO-1 protein expression, HO-1 relative mRNA expression, F4/80 (HO-1-positive macrophages), IL-10 protein, IL-10 relative mRNA expression, CD163 mRNA expression, and Arg-1 mRNA expression and decreased iNOS mRNA expression in stomach tissues. HO-1-positive macrophages are known as M2 macrophages, and the balance between M1 and M2 macrophages is known to be associated with Arg-1 and iNOS. This study showed that the anti-oxidative and anti-inflammatory effects of EA are mediated by regulating the expression of M1 and M2 macrophages, and EA has protective effects for ICC networks through increasing IL-10 expression and decreasing MDA levels (42).

In the intestinal I/R injury mouse model, EA at ST36 reduced the level of IL-6 and TNF-α in serum and decreased mucosal mRNA expression of α7nAChR and NF-κB p65 in the small intestine. In the case of α7nAChR agonist administration, the effects of EA remained the same, whereas in the case of α-BGT injection, which is α7nAChR antagonist, EA effects were reversed. These results indicated that α7nAChR subunit activation and downregulation of NF-κB play an essential role in the anti-inflammatory mechanisms of EA (45). In another intestinal I/R injury model, ST36 EA increased Ki67 proliferation index in intestinal crypt cells, mRNA expressions of stromal-derived factor (SDF)-1, CXCR4, epidermal growth factor (EGF), and EGFR in distal ileum mucosa. Ki67 is a well-known marker of proliferation, and the data in this study showed that EA is a significant proliferation stimulus. EGF and its binding receptor EGFR regulate cell survival and restore the injury of intestinal tissues, and the ligand SDF-1 and its receptor CXCR4 promote the migration of mesenchymal stem cells (MSCs) to the impaired tissues. Also, EA significantly lowered IL-6 and TNF-α in serum through downregulating NF-κB p65 mRNA expression in distal ileum mucosa. This study suggests the ability of EA of regulating the NF-κB signaling pathway and synergizing MSC transplantation as a therapeutic strategy of inflammation (50).

In intra-abdominal adhesion formation model, EA at ST36 decreased TNF-α level, VEGF level, CD31+ expression, and microvessel count in cecal adhesive tissues. In contrast, VGX and α-BGT injection diminished the protective effects of EA. EA might attenuate local inflammation after abdominal surgeries and reduce angiogenesis through inhibiting VEGF and CD31 expressions. This result indicates that the anti-inflammatory mechanism of EA depends on the intact vagus nerve and α7nAChR (71).

In the HS model, EA at ST36 increased glial fibrillary acidic protein (GFAP) expressions and tight junction protein zona occludens (ZO)-1 expression in the intestine and decreased plasma dextran and intestinal TNF-α level. GFAP is a protein marker that represents activation of enteric glial cells (EGCs), which predominantly compose the enteric nervous system (ENS). These showed that EA improved intestinal barrier integrity and reduced intestinal permeability through activating EGCs and increasing ZO-1 expressions. Furthermore, when surgical VGX and α-BGT injection were conducted, EA effects all disappeared. It indicates that EA attenuates gut inflammation through vagus nerve-mediated EGC activation and cholinergic anti-inflammatory mechanisms involving α7nAChR (53).

In the CLP-induced sepsis model, EA at ST36 decreased the circulating d-lactose in serum, which means intestinal permeability, and increased IgA level, percentage of CD3+, γ/δ, CD4+, CD8+ T lymphocytes, and the ratio of CD4+/CD8+ T cells in intestinal mucosa cells. d-Lactose is a product of intestinal bacteria, considered as a biomarker representing high intestinal permeability. This study demonstrates that EA may have beneficial effects on improving the intestinal mucosal immune barrier *via* regulating T lymphocytes (52).

In colitis induced by the TNBS model, EA decreased MPO activity, IL-1β concentration, iNOS expression, and MDA concentration but increased IL-10 concentration in intestine tissues. MPO activity was measured for neutrophil accumulation and inflammation in tissues, and MDA level was measured as an index of lipid peroxidation. The results of this study suggest that EA has immune regulatory and anti-oxidative properties through elevating IL-10 followed by lowering iNOS and inflammatory mediator IL-1β (72).

In the study that used postoperative abdominal adhesion-formed model, EA at ST36 reduced inflammatory mediators including TNF-α level, NO level, and NO synthase (NOS) activity in the cecum, whereas EA with VGX and α-BGT showed less inhibitive effects. This finding indicates that the anti-inflammatory effect of EA is mediated by cholinergic anti-inflammatory cascades by cholinergic neurotransmitter Ach and α7nAChR, and the central nervous system including the vagus nerve (73).

The study that used BALB/c mice cultured with IL-4 revealed that EA at ST36 increased urea production, arginase activity, number of parasite-infected macrophages in peritoneal cells, and IL-4 receptor expression in peritoneal macrophages but decreased NO production in peritoneal cells. This finding demonstrates that ST36 EA induces the generation of alternatively activated macrophages (AAMo), which are anti-inflammatory macrophages stimulated by IL-4 cytokines, through increasing IL-4 responsiveness (74).

In the formaldehyde-induced stomachache model, EA at ST36 increased the absorbance of NOS, acetylcholinesterase (AChE), and vasoactive intestinal peptide (VIP) and decreased calcitonin gene-related peptide (CGRP) in the pyloric sphincter. VIP is known to promote NO releases and induce the relaxation of gastrointestinal smooth muscle, and CGRP is known to be associated with the function of gastrointestinal smooth muscle. This result showed that EA can regulate gastrointestinal motility in the inflammatory pain model, mediated by neurotransmitters of the ENS (75).

### Nervous System

Of the studied 69 articles, 17 articles studied the anti-inflammatory mechanism of acupuncture on ST36 on the nervous system (**Table 3**).

**Table 3:** Anti-inflammatory effect of acupuncture on ST36 in nervous system.

Author	Year	Mouse Model	Acupuncture Method	Target	Inflammatory Markers
Zhao P, et al. (76)	2021	Experimental autoimmune encephalomyelitis (EAE)	2-Hz electroacupuncture (EA)	Spinal cord white matter	Number of inflammatory cells ▽ Percentage area of demyelination ▽
				Brain	T-bet expression, RORrt expression ▽ infiltration of CD4+ T cells ▽
				Hypothalamus	POMC* △ MiR-155 expression ▽
				Cerebral cortex	POMC expression △ MiR-155 expression ▽
Zhang Z, et al. (22)	2021	BALB/c mice with breast tumors	2/15-Hz EA	ChAT+ (choline acetyltransferase-positive) neurons in DMV (dorsal motor nucleus of the vagus)	Expression levels of c-Fos △
Chen Y, et al. (69)	2021	Dextran sulfate sodium (DSS)-induced intestinal inflammation	100-Hz, 25-Hz, 5-Hz EA	S2–3 dorsal root ganglions (DRGs)	TRPV1+ ganglion cell expression (TRPV1-positive area) ▽
Zhao YX, et al. (35)	2020	Pentoxifylline (PTX)-injected mechanical allodynia (PTX-induced neuropathic pain)	10-Hz EA	Lumbar spinal cord	GFAP protein expression ▽ TMEM119 protein expression ▽ TLR4 protein expression ▽ NF-κB p65 protein expression ▽ IL-1β ▽ TNF-α ▽
Li Y, et al. (77)	2019	Complete Freund’s adjuvant (CFA)-induced inflammatory pain	2-Hz EA	Lumbar spinal cord	CX3CL1 expression ▽ Phospho-p38 MAPK level ▽ IL-1β, IL-6, TNF-α ▽
Kim S, et al. (78)	2018	Chronic overlapping pain conditions	Manual acupuncture (MA)	Spinal cord	p-p38 expression ▽ Astrogliosis marker GFAP expression (astrocytes activation) ▽
Lim HD, et al. (48)	2016	Lipopolysaccharide (LPS) administration endotoxemia	MA 1-Hz EA	Brain tissue (brain stem transverse section)	Number of c-Fos cells ▽
Huang CP, et al. (79)	2013	Carrageenan- and CFA-induced inflammatory pain	2-Hz EA	L3–L5 DRG neurons	Nav 1.7, Nav 1.8 sodium channels expression ▽ Nav 1.7, Nav 1.8 protein level ▽ TTX-R* sodium currents △
Chen WH, et al. (80)	2012	Carrageenan- and CFA-induced inflammatory pain	2-Hz EA	DRGs	TRPV1, TRPV4 expression ▽
Zhang Z, et al. (81)	2012	Carcinoma cell inoculation (cancer pain)	2-Hz EA	L3–5 DRG	TRPV1 protein expression ▽ TRPV1 mRNA expression ▽
Xu X, et al. (75)	2012	Formaldehyde-induced stomachache (visceral pain)	4/16-Hz EA	Submucosal plexus and myenteric nerve plexus of the sphincter	NOS-positive neuron perikarya, enzymatic activity △ Number of CGRP-positive nerve fibers ▽ Number of AchE-positive perikarya and enzymatic activity △ Number of VIP-positive nerve fibers △
Chen WH, et al. (82)	2011	Carrageenan- and CFA-induced inflammation	2-Hz EA	Lumbar DRGs	ASIC3* protein ▽ ASIC3 mRNA ▽
Gim GT, et al. (63)	2011	Neuropathic surgery	2-Hz EA	S1 spinal cord	Microglial activation (number of OX-42+ cell) ▽ OX-42 expression ▽ Astrocytic activation (number of GFAP+ cell) ▽ GFAP expression ▽ MMP-9, MMP-2 expression ▽ Proinflammatory cytokines (TNF-α, IL-6, IL-1β) ▽
Liu YM, et al. (64)	2010	Experimental autoimmune encephalitis	1-Hz EA	Spinal cords (neural tissue)	Cellular infiltrates ▽
				Hypothalamus	ACTH △
Yang EJ, et al. (83)	2010	hG93ASOD1 mouse (inherited amyotrophic lateral sclerosis (ALS))	2-Hz EA	Lumbar spinal cord (L4–5) ventral horn region anterior horn	Iba1 protein expression ▽ Number of Iba1-positive cells ▽ MAP2 expression △ Cell counts of NeuN-positive cells △ TNF-α-IR expression (number of TNF-α-positive cells) ▽ Active AKT, phospho-ERK expression △ Phospho-p38 protein, active caspase-3 expression ▽
				Facial nucleus of the brain stem	Iba1 protein expression ▽ Iba1-immunoreactive cells ▽ MAP2 expression △ Cell counts of NeuN-positive cells △ TNF-α-IR expression (number of TNF-α-positive cells) ▽ Active AKT, phospho-ERK expression △
Kim HW, et al. (84)	2007	Zymosan-induced inflammation	1-Hz EA	Intermediolateral (IML) cell column of thoracic spinal cord	Number of ChAT and Fos double-labeled neurons △
Sung HJ, et al. (85)	2004	Neuropathic pain model	2-Hz EA	Hypothalamus	IL-1b converter precursor, porphobilinogen deaminase, calretinin, phosphorylase B kinase x catalytic chain, glial fibrillary acidic protein, Ras-related protein RAB-15, stress-activated protein kinase JNK3, IL-18 precursor, ubiquinone biosynthesis protein COQ7, protein-tyrosine phosphatase a precursor, proteasome component C8 △ Phosphatidylinositol transfer protein a isoform, cell division protein kinase 4, choline phosphate cytidylyltransferase, neuronal kinesin heavy chain, α-enolase, vitamin D binding precursor, dihydropyrimidinase-related protein-2, myosin regulatory light chain2, somatotropin precursor, fructose-biphosphate aldolase C (brain) ▽ β-Adrenergic receptor kinase1, kinesin light chain, 14-3-3x, rac-B-serine/theronine kinase, tyrosine-protein kinase Lyn △

*POMC, pro-opiomelanocortin; TTX-R, tetrodotoxin-resistant; ASIC3, acid-sensing ion channel 3.

In the experimental autoimmune encephalomyelitis (EAE) model, EA at ST36 decreases the number of inflammatory cells and demyelination area in spinal cord white matter. EA downregulates the infiltration of CD4+ T cells and the expressions of transcription factors of Th1 and Th17 cells, T-bet, and ROR-γt, in the brain. In spleen lymphocytes, EA reduces the proportion of IFN-γ, IL-17, and IL-4 expressions, which are Th1, Th17, and Th2 cytokines, respectively. Additionally, EA shows anti-inflammatory effects on the central nervous system through inhibiting the miR-155 expression and promoting pro-opiomelanocortin (POMC) expression in both the hypothalamus and cerebral cortex. Meanwhile, the reduction of T-bet, ROR-γt, IFN-γ, and IL-17 expressions was reversed by miR-155 mimic reagents, indicating that alleviation effects on EAE of EA were mediated by inhibition of miR-155 (76).

In the pentoxifylline (PTX)-injected mechanical allodynia model, ST36 EA decreased the expression of GFAP, TMEM119, TLR4, NF-κB p65, IL-1β, and TNF-α proteins in the lumbar spinal cord and IL-1β and TNF-α in serum. This reduction of proinflammatory cytokines is mediated by downregulating the glial activity and restraining the activation of the TLR4/NF-κB signaling pathway (35).

In the CFA-induced inflammatory pain model, EA at ST36 reduced CX3CL1 expression and phospho-p38 MAPK, IL-1β, IL-6, and TNF-α levels in the lumbar spinal cord, whereas the injection of CX3CL1 reversed these analgesic effects. EA inhibited the production of CX3CL1 cleaved form combined with its receptor, CX3CR1, and prevented p38 MAPK phosphorylation and release of inflammatory pain-related cytokines. Ultimately, EA alleviated inflammatory pain *via* modulating the downstream pathway of CX3CL1/CX3CR1-p38 MAPK signaling (77). In another study that used carrageenan- or CFA-induced inflammatory pain model, Nav 1.7 and Nav 1.8 sodium channel expressions and protein levels were decreased in L3–5 DRG neurons, and tetrodotoxin-resistant (TTX-R) sodium currents were increased after EA treatment. Nav 1.7 protein is largely expressed in free nerve endings of C-fiber, delivering nociceptive information. EA at ST36 showed analgesic effects against inflammatory pain by preventing the overexpression of Navs (79). Moreover, ST36 EA decreased expression of TRPV1 and TRPV4 in DRGs in carrageenan- and CFA-induced inflammatory pain models. TRPV4 and TRPV1 are both cation channels and are crucially involved in hyperalgesia. This study explains that the mechanism of the anti-nociceptive effect of EA on inflammatory pain is the activation of A1R, which is a G-protein-coupled receptor (GPCR), decreasing adenylyl cyclase activities and activating pertussis toxin-sensitive Gi protein. At last, EA inhibits protein kinase A (PKA) activity and downregulates TRPV1 and TRPV4 expressions (80). Furthermore, in the carrageenan- and CFA-induced inflammation model, ST36 EA reduced the expressions of acid-sensing ion channel 3 (ASIC3) and ASIC3 mRNA in lumbar DRGs. This result indicated that EA is able to attenuate inflammatory hyperalgesia through the downregulation of ASIC3 proteins (82).

In chronic overlapping pain conditions, ST36 MA reduced p-p38 and GFAP+ astrocyte expression in the spinal cord. GFAP astrocytes are the immune cells located in CNS, playing a critical role in nerve injury or neuroinflammation-induced pain. In this study, acupuncture ameliorated mechanical hypersensitivity by suppressing key factors in the catecholamine signaling pathway (78).

In the LPS administration endotoxemia model, both MA and EA at ST36 decreased the number of c-Fos cells in brain tissues and TNF-α in serum and decreased TNF-α mRNA expression and TNF-α signal intensity in the spleen. TNF-α is known to induce c-Fos expressions nearby dorsal vagal complex (DVC) neurons by regulating vagal afferent-releasing neurotransmitters. The decline of c-Fos cells following acupuncture was reversed by the administration of CNQX, which is an AMPA glutamate receptor blocker, and PPADS, which is a purinergic receptor antagonist. These data showed that glutaminergic and purinergic receptors in the DVC are activated by acupuncture signals resulting in vagal modulation. Also, the decreased TNF-α level in the spleen was elevated by the splenic neurectomy and the VGX, indicating that the modulatory effect of TNF-α production *via* acupuncture is triggered by activated splenic nerve through vagus nerve activation. These results suggest that acupuncture therapeutic effects against inflammation are mediated by promoting synaptic transmissions in the vagus nerve and TNF-α signaling pathways involving the splenic nerve (48).

In the carcinoma cell inoculation model, ST36 EA relieved the cancer-induced pain by suppressing the expression of TRPV1 protein and mRNA in the L3–5 DRG. This study speculates that this EA effect is mediated by the release of adenosine and the activated adenosine A1 receptor (81).

In the neuropathic surgery model, EA at ST36 decreased the number of OX-42+ cells, the expression of OX-42, the number of GFAP+ cells, and the expression of GFAP and eventually reduced the level of proinflammatory cytokines such as TNF-α, IL-6, and IL-1β in the S1 spinal cord. Also, EA upregulated the concentration of IgG in serum, one of the inflammation-associated factors, and IgG promoted neurological recovery resulting in the suppression of MMP-9 and MMP-2 expressions in the S1 spinal cord. The data in this study showed that EA inhibits inflammation and spinal glial activation through attenuating microglial and astrocytic activation and controlling MMPs (63). Another study that used the neuropathic pain model showed that multiple signaling transduction pathways are involved in the anti-inflammatory effect of EA at ST36. EA restored the decreased level of IL-1β converter precursor, porphobilinogen deaminase, calretinin, phosphorylase B kinase χ catalytic chain, GFAP, Ras-related protein RAB-15, stress-activated protein kinase JNK3, IL-18 precursor, ubiquinone biosynthesis protein COQ7, protein-tyrosine phosphatase α precursor, proteasome component C8, β-adrenergic receptor kinase1, kinesin light chain, 14-3-3 protein χ, AKT2, and tyrosine-protein kinase Lyn after neuroinflammation to the normal range. Also, EA restores the increased level of phosphatidylinositol transfer protein α isoform, cell division protein kinase 4, choline phosphate cytidylyltransferase, neuronal kinesin heavy chain, α-enolase, vitamin D binding precursor, dihydropyrimidinase-related protein-2, myosin regulatory light chain2, somatotropin precursor, and fructose-biphosphate aldolase C (85).

In the hG93A SOD1 mouse model, which inherited amyotrophic lateral sclerosis (ALS), EA at ST36 decreased Iba1 protein expression, the number of Iba1-positive cells, and the number of TNF-α positive cells in both the lumbar spinal cord and brain stem and decreased phospho-p38 protein and active caspase-3 expression in the lumbar spinal cord. This phenomenon shows that EA suppressed neuroinflammation by blocking the activation of p38 MAPK downstream pathway and TNF-α signaling because activated caspase-3 induces microglial cell activation, which leads to the production of neuroinflammatory cytokines. Also, EA increased MAP2 expression, cell counts of NeuN-positive cells, active-AKT expression, and phospho-ERK expression in both the spinal cord and brain stem, indicating that EA can inhibit the death of neuronal cells and delay the disease progress. The anti-inflammatory effect of EA is derived from regulating the activation of microglial cells and cell survival signaling pathways (83).

In the zymosan-induced inflammation model, ST36 EA increased the number of choline acetyltransferase (ChAT) and the expression of Fos double-labeled neurons in the intermediolateral (IML) cell column of the thoracic spinal cord. Also, EA suppressed the leukocyte migrations induced by zymosan, but adrenalectomy (ADX), 6-hydroxydopamine (6-OHDA) treatment, and propranolol (PRO) treatment reversed the suppressive effects of EA. These results suggest that sympatho-adrenal medullary axis, catecholamines, and β-adrenoceptors possibly play an important role in the anti-inflammatory effects of EA (84).

### Others

Of the total articles studied in this analysis, 30 articles were analyzed in **Table 4**. **Table 4** shows how acupuncture at ST36 affects other organs or tissues except for body fluids and the nervous and digestive systems. This analysis includes the spleen, lung, liver, ST36 localized tissues, knee joint, ankle joint, muscle tissues, foot tissues, ear tissues, cardiac tissues, skin, adipose tissues, flap tissues, and tumor tissues.

**Table 4:** Anti-inflammatory effect of acupuncture on ST36 in others.

Author	Year	Mouse Model	Acupuncture Method	Target	Inflammatory Markers
Yang F, et al. (86)	2021	Adjuvant-induced arthritics (AIA)	MA	Right ankle homogenate	TNF-α, IL-1β ▽ IL-1β, TNF-α, IL-18, IL-6, IL-7, IL-12, IL-2, IL-4, IL-10, Il-13, IL-5, RANTES, G-CSF, EPO ▽ IL-1α, IFN-γ, IL-17, MCP-1, MIP-1α, MIP-3α, CXCL1, GM-CSF, M-CSF, VEGF △
				Right ankle joint	Population of CD45+CD11b+ macrophages ▽ Proportion of CD86+CD206− (M1 macrophages) ▽ Proportion of CD86−CD206+ (M2 macrophages) △ Ratio of M1/M2 population △ IL-1β protein level ▽
Zhao P, et al. (76)	2021	EAE	2-Hz EA	Spleen lymphocyte	Proportion of IFN-γ expression ▽ Proportion of IL-17 expression ▽ Proportion of IL-4 expression △
Zhang Z, et al. (22)	2021	BALB/c mice with breast tumors	2/15-Hz EA	Tumor tissue	IL-1β protein expression, TNF-α protein expression ▽ IL-10 protein expression △ Proportions of CD8+ cells △ Proportions of NK cells △ Expression of perforin protein, granzyme B protein △ Proportions of MDSCs (CD11b+Gr-1+) ▽ Expression levels of COX-2* protein, Arg-1 protein ▽
				Spleen T cells (CD4+ T cells, CD8+ T cells)	Expression of CD25 △
Zhang L, et al. (27)	2021	PDL-induced pancreatitis	2/15-Hz EA	Lung tissue	MPO expression ▽
Lim HD, et al. (31)	2020	ConA model of hepatitis	MA 1-Hz, 5-Hz EA	Liver tissue	CD68 protein ▽ CD11b protein ▽ TNF-α ▽ p-ERK1/2* expression ▽
Xie LL, et al. (32)	2020	DIO-KOA	30-Hz EA	Knee joint (arthrodial cartilage)	MMP-1, MMP-13 ▽ TLR4, NF-κB p65, NF-κB P-p65 ▽
Zhang K, et al. (87)	2020	CFA-induced inflammation model	MA	ST36 tissue	MCP-1, CXCL1, MIP-3a, MIP-1a, RANTES △ IL-1β, IL-1α, IL-6, IL-2, IL-12, IL-13 △ IL-18, IL-10, CRP, IFN-γ ▽ GM-CSF ▽ G-CSF, M-CSF △ Percentage of CD45+CD11b+CD68+ cells △
Wang Z, et al. (39)	2019	DNFB-induced ACD	2-Hz EA	Ear tissue	Number of mast cells ▽
Chen T, et al. (40)	2019	LPS-induced inflammation	2-Hz EA	Spleen mononuclear cell	Ca^2+^ influx ▽ TLR4 expression ▽ CB2R expression △
Wang Z, et al. (70)	2018	DNFB-induced ACD	2-Hz EA	Ear tissue	Number of mast cells. mast cell infiltration ▽ IL-33 ▽
Zhang K, et al. (41)	2018	CFA-induced inflammation	MA	ST36 tissue	TSH, corticosterone, FSH, melatonin, GRO/KC, IL-1β, IL-6, M-CSF △
				Hind footpad tissue	BDNF, RANTES, M-CSF ▽ CRH △
Zhang L, et al. (43)	2018	Myocardial injury sepsis model	2/100-Hz EA	Cardiac tissue	TNF-α content ▽ NO content ▽ MPO content ▽ Moisture rate ▽
Chen L, et al. (44)	2017	SD rats	2-Hz, 15-Hz EA	Spleen	IL-2, IL-17 △ CD4 expression △ Ca^2+^ concentration △
				ST36 tissue	IFN-γ level △
Wang LR, et al. (21)	2017	McFarlane flap-established model	10-Hz EA	Flap tissue	Percentage of survival area △ Mean vessel density △ VEGF* expression △ SOD* activity △ Mean MDA level ▽
Wang Z, et al. (46)	2017	DNFB-induced ACD	2-Hz EA	Ear tissue	Inflammatory cell infiltration ▽ Inflammatory cell densities (numbers) in the dermis ▽ Th1-type cytokines (IFN-γ, TNF-α, IL-1β) ▽ Th2-type cytokines (IL-4, IL-5, IL-10) ▽
				Spleen lymphocyte	CD4+IFN-γ+ T cells ▽ CD4+IL-4+ T cells ▽ p-ERK protein, p-JNK protein ▽ p-p38 protein ▽
				ST36 tissue	IFN-γ, IL-4 ▽ IL-10 △ IL-10+ macrophages percentage △
Wang Z, et al. (47)	2017	OVA-induced DTH	2-Hz EA	Spleen lymphocyte	IFN-γ, T-bet ▽ T-bet/GATA-3 (Th1/Th2) ratio ▽
				Footpad tissue	Number of inflammatory cell ▽ Th1-type cytokine (IFN-γ, TNF-α) ▽
Lim HD, et al. (48)	2016	LPS administration endotoxemia	MA 1-Hz EA	Spleen	TNF-α mRNA expression ▽ TNF-α signal intensity ▽
Li H, et al. (88)	2015	Third lumbar vertebrae transverse process syndrome	2/100-Hz EA	Third lumbar vertebrae muscle tissue	IL-1β mRNA expression ▽ TNF-α mRNA expression ▽ iNOS mRNA expression ▽
Song XM, et al. (54)	2015	TBSA scald subjected TEM	3-Hz EA	Lung tissue (bronchial epithelium, luminal surface)	HMGB-1 expression (mean ODs*) ▽ HMGB-1 mRNA expression ▽ Inflammatory cellular infiltration ▽
Wen CK, et al. (51)	2015	Obese leptin-deficient mice	2-Hz EA	White adipose tissue	Mean area of adipocyte ▽ HIF-1a protein ▽ HIF-1a, VEGFA, S1c2a1, GPX1 mRNA ▽ F4/80, TNF-α, MCP-1, IL-6 mRNA ▽ F4/80 protein, NF-κB protein ▽ IkBa protein △
Villegas-Bastida A, et al. (55)	2014	CLP-induced polymicrobial sepsis model	30-Hz EA	Spleen	Nuclear fraction NF-κB p65 activity ▽
Wen CK, et al. (56)	2014	HFD-induced obesity model	10-Hz EA	Adipose tissue	SREBP1c, ACC, FAS, SCD1 ▽ Area neutrophil+, area CD11b+ ▽ Area F4/80+ ▽ F4/80 mRNA, TNF-α mRNA, MCP-1 mRNA, CD68 mRNA, IL-6 mRNA ▽
Wu SY, et al. (89)	2014	CFA-injected inflammatory pain model	MA	ST36 tissue (muscle)	TRPV1, TRPV4, ASIC3, PanX1, Cx43, P2Y1 △ PGP 9.5 ▽
				ST36 tissue (subcutaneous loose connective tissue)	TRPV1, TRPV4, ASIC3, Cx43, P2Y1, P2Y2 △ PGP 9.5, PanX1 ▽
Geng WY, et al. (62)	2013	COPD	2/60-Hz EA	Lung	Bronchi, bronchiole obstruction on lung histopathological sections ▽ RL* ▽ CL* △
Hu S, et al. (61)	2013	Intestine-ischemia (I/R) model	2/100-Hz EA	Lung	TNF-α, IL-8 ▽
				Liver	TNF-α, IL-8 ▽
Smeester BA, et al. (90)	2012	Tumor-induced hyperalgesia	4-Hz EA	Osteosarcoma tumor tissue	Pixel NIMP-R14-positive (neutrophils) ▽ Pixel MOMA-2-positive (macrophages) ▽
				Fibrosarcoma tumor microperfusate sample	PGE2* ▽
Jiang JH, et al. (91)	2011	hSOD1G93A transgenic mice (inherited ALS)	2-Hz EA	Lung	Iba-1, TNF-α protein ▽ Number of Iba-1, TNF-α immunoreactive cell ▽ IL-6 protein, NF-κB protein expression ▽ p-AKT, p-ERK expression △
Kim HW, et al. (92)	2008	Carrageenan-induced inflammation	1-Hz EA 120-Hz EA	Subcutaneous tissue of the paw	MPO activity ▽
Yim YK, et al. (67)	2007	CIA	2-Hz EA	Knee joint	Histological changes ▽
Moon PD, et al. (93)	2007	Passive cutaneous anaphylaxis (PCA) model	2-Hz EA	Dorsal skin (tissue protein)	β-Hexosaminidase activity inhibition rate △ IL-6, TNF-α secretion inhibition rate △ NF-κB DNA-binding activity inhibition rate △

*COX-2, cyclooxygenase-2; p-ERK1/2, phosphor-ERK1/2; VEGF, vascular endothelial growth factor; SOD, superoxide dismutase; ODs, optical densities; RL, lung resistance; CL, lung compliance; PGE2, prostaglandin E2.

In the adjuvant-induced arthritis (AIA) model, ST36 MA showed remarkable anti-inflammatory effects on ankle joints. MA reduced pathological score, TNF-α level, and IL-1β level in right ankle homogenate. Twenty-four inflammation-associated genes were detected on days 1, 7, 15, and 21 after MA treatment, and the outcome data on day 21 are summarized in **Table 4**. It suggests that acupuncture at ST36 has regulatory effects on cytokines related to innate and adaptive immunity but has insignificant effects on chemokines and growth factors. Additionally, acupuncture decreased the proportion of M1 macrophages and increased M2 macrophages, showing that MA has anti-inflammatory actions *via* modulating macrophage polarization, resulting in the downregulation of IL-1β protein levels (86).

In the model of breast tumor mice, EA significantly reduced the level of inflammatory cytokines including IL-1β and TNF-α and increased the level of the anti-inflammatory cytokine, IL-10, in serum. Likewise, EA reduced the expressions of IL-1β protein and TNF-α protein and increased the expression of IL-10 protein in tumor tissues. EA also induced the increase of CD8+ T cells and NK cells in the blood and spleen. In local tumor tissues, EA obviously increased NK cell proportion and slightly improved CD8+ T cell proportion and noticeably increased the expression of perforin protein and granzyme B protein. This indicates that EA enhances anti-tumor immune responses *via* the perforin-related cytolytic pathway. Moreover, accumulated CD11b+Gr-1+ cells by breast cancer, in the blood, spleen, and tumor tissues markedly decreased after EA intervention. Arg-1 protein and COX-2 protein, which are crucial mediators of myeloid-derived suppressor cells (MDSCs) that suppress the function of T cells and NK cells, were detected. The results showed that EA reduces the expressions of Arg-1 and COX-2 and attenuates the immunosuppressive capacity of MDSCs. To investigate whether EA affects the activation level of T cells, CD25, which predominantly exists on activated T cells, was detected in spleen T cells, and its expression also increased after EA intervention. EA leads to c-Fos expressions in ChAT-positive neurons in the dorsal motor nucleus of the vagus, and these ameliorating effects of EA on tumor growth were abolished by subdiaphragmatic VGX. It demonstrates that EA alleviates tumor growth by activating vagal outputs (22).

In the concanavalin A (ConA) model of hepatitis, both MA and EA attenuated TNF-α production in serum, but when VGX was conducted, the production of TNF-α elevated. Acupuncture at ST36 also decreased CD68 protein, CD11b protein, TNF-α, and phospho-ERK1/2 (p-ERK1/2) expression in liver tissues. Since activated p-ERK1/2 is known to induce TNF-α production in hepatic Kupffer cells, this study investigated CD68 and CD11b proteins as marker proteins of activated Kupffer cells. These results showed that the anti-inflammatory effect of acupuncture is mediated by vagus nerve activity modulating ERK1/2-STAT3 signaling cascade linked to the cholinergic anti-inflammatory mechanism (31).

In the myocardial injury of sepsis model, EA at ST36 lowered CK-MB content in plasma and TNF-α, NO, and MPO contents in cardiac tissues. Meanwhile, the excision of the bilateral ventral vagus nerve caused the depletion of anti-inflammation and cardioprotective effects of EA. This observation indicates that the mechanism of EA at ST36 is related to the cholinergic anti-inflammatory pathway, and the level of proinflammatory cytokines is regulated by the balance of the sympathetic adrenergic nerve and parasymptomatic cholinergic nerve (43).

In rats, EA at ST36 increased the level of IFN-γ, IL-2, and IL-17 and decreased the level of Ca^2+^ in serum. Also, EA increased IL-2, IL-17, and CD4 expression and Ca^2+^ concentration in the spleen and IFN-γ level in ST36 tissues. This study found that EA stimulation on ST36 activated TRPV channels including TRPV1 and TRPV4 in splenic T cells, which are non-selective Ca^2+^ channels. The activation of TRPV channels increased Ca^2+^ influx into spleen cells, differentiated and activated CD4+ T cells in the spleen, and eventually increased the level of IFN-γ, IL-2, and IL-17 (44).

In McFarlane flap-established model, EA at ST36 increased the percentage of survival area, mean vessel density (MVD), VEGF expression, and superoxide dismutase (SOD) activity and decreased the mean MDA level in flap tissues. ST36 EA enhances flap survival *via* promoting VEGF expressions and MVD. Also, EA inhibits lipid peroxidation, removes oxygen-free radicals, and displays anti-oxidant effects, through increasing SOD activity and decreasing MDA levels (21).

In the DNFB-ACD model, EA at ST36 decreased proinflammatory cell infiltration and proinflammatory cell densities in the dermis and Th1-type cytokines including IFN-γ, TNF-α, IL-1β, and Th2-type cytokines such as IL-4, IL-5, and IL-10 in ear tissues. It indicates that EA demonstrably attenuated ear swelling and inhibited proinflammatory cytokine productions through regulating Th1/Th2 balance. Moreover, EA reduced CD4+IFN-γ+ T cells, CD4+IL-4+ T cells, p-ERK protein, P-JNK protein, and p-p38 protein in spleen lymphocytes. EA downregulated IFN-γ and IL-4 and upregulated IL-10 and IL-10+ macrophage percentage in ST36 tissues and decreased the level of IgE in serum. It shows that decreased IFN-γ and increased IL-10 influence the development of immune responses. These observations suggest that EA treatment triggers the production of IL-10 in local acupoint and suppresses the activation of p38 MAPK, a key mediator of persistent inflammation (46).

In the ovalbumin (OVA)-induced delayed-type hypersensitivity (DTH) model, ST36 EA decreased the level of IgE and OVA-specific IgG in serum and decreased IFN-γ, T-bet, and T-bet/GATA-3 (Th1/Th2) ratio in spleen lymphocyte. Also, EA reduced the number of inflammatory cells, Th1-type cytokines such as IFN-γ, and TNF-α in footpad tissues. To sum up, EA restored the balance of Th1 and Th2 by curbing the differentiation of Th1 cells (47).

In the third lumbar vertebrae transverse process syndrome model, EA at ST36 decreased mRNA expressions of IL-1β, TNF-α, and iNOS in third lumbar vertebrae muscle tissues. This result shows that EA can exert anti-inflammatory effects through downregulating the concentration of main proinflammatory cytokines and iNOS, which generates NO and inflammatory responses (88).

In the obese leptin-deficient mice model, EA at ST36 decreased the level of insulin, glucose, triglyceride, total cholesterol, and inflammatory cytokines including TNF-α, IL-6, and IL-1β in serum. EA decreased the mean area of adipocyte, hypoxia-inducible factor (HIF)-1α protein, and the mRNA expressions of hypoxia-related genes such as HIF-1α, VEGFA, S1c2a1, and GPX1 in white adipose tissues. Also, EA reduced a macrophage cell marker F4/80, TNF-α mRNA, MCP-1 mRNA, IL-6 mRNA, F4/80 protein, and NF-κB protein and increased IκBα protein in adipose tissues. The therapeutic effect in obese subjects of EA is the alleviation of obesity-induced inflammation through suppressing HIF-1α signaling and restraining the NF-κB signaling pathway (51).

In the high-fat diet-induced obesity model, EA lowered alanine transaminase (ALT) and aspartate transaminase (AST) levels, indicating that EA potentially prevents the damage of hepatocytes. Also, EA decreased total cholesterol, triglyceride, free fatty acid, TNF-α, IL-1, and IL-6 in plasma. It is a result of EA reducing the expression of lipogenic genes including SREBP-1c, ACC, FAS, and SCD1 in adipose tissues. Furthermore, EA ameliorated obesity-associated inflammatory responses through decreasing area neutrophil+, area CD11b+, area F4/80+, F4/80 mRNA, TNF-α mRNA, MCP-1 mRNA, CD68 mRNA, and IL-6 mRNA in adipose tissues. The regulatory effect of EA against inflammation in adipose tissues is mediated by suppressing the lipogenic pathway and reducing macrophage infiltration and inflammatory mediators (56).

In the tumor-induced hyperalgesia model, EA at ST36 decreased pixel NIMP-R14-positive neutrophils in osteosarcoma tumor tissues, pixel MOMA-2-positive macrophages, and prostaglandin E2 (PGE2) in fibrosarcoma tumor micro-perfusate sample. These data demonstrated that EA may exert beneficial effects on relieving tumor-induced nociception and tumor-associated inflammation, through regulating PGE2 production and the density of neutrophils and macrophages at the tumor site (90).

In the ALS model, a study used hSOD1G93A transgenic mice, and EA at ST36 decreased protein expressions of Iba-1 and TNF-α, the number of inflammatory cells including Iba-1 and TNF-α immunoreactive cell, and the inflammatory protein expressions such as IL-6 and NF-κB in the lung. Also, EA increased cell survival protein expressions of p-AKT and p-ERK in the lung. These data demonstrated that the mechanism of modulating inflammation by EA in the pulmonary system is suppressing the activity of NF-κB and reducing Iba-1 expression secreting TNF-α (91).

In the carrageenan-induced paw inflammation model, both LEA and HEA reduced MPO activity in the subcutaneous tissues of the paw. However, ADX, 6-OHDA treatment, and PRO treatment diminished the EA effects on MPO activity, whereas a corticosterone receptor antagonist RU-486 administration did not affect EA treatments. 6-OHDA is a selective neurotoxin that targets catecholaminergic neurons destroying sympathetic nerve endings, and it influenced only LEA effects. This result demonstrates that LEA produces its suppressive effects through activating sympathetic post-ganglionic nerve (SPGN) fibers. Also, HEA effects were significantly abolished by ADX, indicating that HEA performs its anti-inflammatory effect *via* activating the sympatho-adrenal axis. Since PRO treatment, as a β-adrenoceptor antagonist, remarkably blocked inhibitive effects of EA, we can expect that both LEA and HEA stimulations are mediated by β-adrenoceptors (92).

In the passive cutaneous anaphylaxis (PCA) model, EA at ST36 increased the inhibition rates of β-hexosaminidase activity, IL-6 secretion, TNF-α secretion, and NF-κB DNA-binding activity but did not change NF-κB/RelA protein expressions in tissue proteins. This study suggests modulatory effects of EA on NF-κB activation (93).

In CFA-induced inflammation model, it showed how MA at ST36 works at sensory receptors and inflammation-related cytokines within the ST36 acupoint. MA at ST36 increased MCP-1, CXCL1, MIP-3α, MIP-1α, RANTES, IL-1β, IL-1α, IL-6, IL-2, IL-12, IL-13, granulocyte colony-stimulating factor (G-CSF), and M-CSF and decreased IL-18, IL-10, CRP, IFN-γ, GM-CSF, and percentage of CD45+CD11b+CD68+ cells in ST36 acupoint tissues. This study revealed the key changes of immune-related cells, cytokines, chemokines, and macrophages caused by MA and defined that MA effects are mediated by regulating cell–cell communication (CCC) networks (87). In another CFA-injected inflammatory pain model, ST36 MA increased TRPV1, TRPV4, ASIC3, PanX1, Cx43, and P2Y1 and decreased PGP 9.5 in muscle tissues at ST36. Similarly, MA increased TRPV1, TRPV4, ASIC3, Cx43, P2Y1, and P2Y2 and decreased PGP 9.5 and PanX1 in subcutaneous loose connective tissues at ST36. However, the injection of capsaicin, which is a TRPV1 agonist, replicated the pain-relieving effects of acupuncture. This finding demonstrates that TRPV1 activation might play an important role in analgesic effects, by transmitting acupuncture signals to nerve terminals *via* calcium wave propagation (CWP), which means downstream sensing pathways associated with calcium influx and ATP release (89).

## Conclusion

In this study, we analyzed animal literatures that investigated how acupuncture treatment at ST36 changes inflammation-associated mediators in various pathological models. Acupuncture at specific acupoint ST36 showed anti-inflammatory properties by regulating inflammation-related genes in body fluids, the digestive system, the nervous system, and other tissues or organs.

It revealed that the anti-inflammatory effects of acupuncture at ST36 depend on several mechanisms including vagus nerve activity, splenic nerve, MAPK signaling pathway, TLR4/NF-κB signaling, c-Kit signaling pathway, cholinergic anti-inflammatory pathway, NEI network, activation of parasympathetic efferent pathway, NGF/TrkA/TRPV1 peripheral afferent pathway, CX3CL1 signaling pathway, NLRP3/IL-1β pathway, CB2R-p38 signaling pathway, inhibition of miR-155, and regulation of macrophage polarization.

With these results, our study will help to reveal potential mechanisms of acupuncture and inform future research related to the role of acupuncture treatment at ST36 as inflammation medications. This article is also expected to contribute to further acupuncture experimental and clinical trials. We believe that this study is worthy of the fundamental research on the inflammatory inhibition effects of acupuncture treatment.

## Author Contributions

J-EO searched the databases and extracted the data. S-NK designed and supervised the study. J-EO and S-NK analyzed the data and wrote the paper.

## Funding

This work was supported by the National Research Foundation of Korea funded by the Korean government (MSIT) (NRF-2020R1C1C1004107) and from the Ministry of Health & Welfare through the Korea Health Industry Development Institute (KHIDI) (grant no. HF21C0018).

## Conflict of Interest

The authors declare that the research was conducted in the absence of any commercial or financial relationships that could be construed as a potential conflict of interest.

## Publisher’s Note

All claims expressed in this article are solely those of the authors and do not necessarily represent those of their affiliated organizations, or those of the publisher, the editors and the reviewers. Any product that may be evaluated in this article, or claim that may be made by its manufacturer, is not guaranteed or endorsed by the publisher.

## References

[1] Papila KB Sozer V Cigdem KP Durmus S Kurtulus D Papila C. Circulating Nuclear Factor-Kappa B Mediates Cancer-Associated Inflammation in Human Breast and Colon Cancer. J Med Biochem (2021) 40:150–9. doi: 10.5937/jomb0-27128 PMC7982282 33776564

[2] Shapouri-Moghaddam A Mohammadian S Vazini H Taghadosi M Esmaeili SA Mardani F. Macrophage Plasticity, Polarization, and Function in Health and Disease. J Cell Physiol (2018) 233:6425–40. doi: 10.1002/jcp.26429 PMC13482560 29319160

[3] Caminero F Iqbal Z Tadi P. Histology, Cytotoxic T Cells. In: StatPearls. Treasure Island FL: StatPearls Publising (2021). 32644705

[4] Minihane AM Vinoy S Russell WR Baka A Roche HM Tuohy KM. Low-Grade Inflammation, Diet Composition and Health: Current Research Evidence and Its Translation. Br J Nutr (2015) 114:999–1012. doi: 10.1017/S0007114515002093 26228057 PMC4579563

[5] Rodrigues EB Farah ME Bottos JM Bom Aggio F. Nonsteroidal Anti-Inflammatory Drugs in the Treatment of Retinal Diseases. Dev Ophthalmol (2016) 55:212–20. doi: 10.1159/000431197 26502088

[6] Yousefifard M Zali A Zarghi A Madani Neishaboori A Hosseini M Safari S. Non-Steroidal Anti-Inflammatory Drugs in Management of COVID-19; A Systematic Review on Current Evidence. Int J Clin Pract (2020) 74:e13557. doi: 10.1111/ijcp.13557 32460369 PMC7267090

[7] Li D Gupta P Sgaglione NA Grande DA. Exosomes Derived From Non-Classic Sources for Treatment of Post-Traumatic Osteoarthritis and Cartilage Injury of the Knee: *In Vivo* Review. J Clin Med (2021) 10:1–13. doi: 10.3390/jcm10092001 PMC8124969 34066986

[8] Tsujimoto S Mokuda S Matoba K Yamada A Jouyama K Murata Y. The Prevalence of Endoscopic Gastric Mucosal Damage in Patients With Rheumatoid Arthritis. PloS One (2018) 13:e0200023. doi: 10.1371/journal.pone.0200023 29985937 PMC6037345

[9] Voiriot G Philippot Q Elabbadi A Elbim C Chalumeau M Fartoukh M. Risks Related to the Use of Non-Steroidal Anti-Inflammatory Drugs in Community-Acquired Pneumonia in Adult and Pediatric Patients. J Clin Med (2019) 8:1–10. doi: 10.3390/jcm8060786 PMC6617416 31163625

[10] Sriuttha P Sirichanchuen B Permsuwan U. Hepatotoxicity of Nonsteroidal Anti-Inflammatory Drugs: A Systematic Review of Randomized Controlled Trials. Int J Hepatol (2018) 2018:5253623. doi: 10.1155/2018/5253623 29568654 PMC5820561

[11] Wongrakpanich S Wongrakpanich A Melhado K Rangaswami J. A Comprehensive Review of Non-Steroidal Anti-Inflammatory Drug Use in The Elderly. Aging Dis (2018) 9:143–50. doi: 10.14336/AD.2017.0306 PMC5772852 29392089

[12] Lucas GNC Leitao ACC Alencar RL Xavier RMF Daher EF Silva Junior GBD. Pathophysiological Aspects of Nephropathy Caused by Non-Steroidal Anti-Inflammatory Drugs. J Bras Nefrol (2019) 41:124–30. doi: 10.1590/2175-8239-jbn-2018-0107 PMC6534025 30281062

[13] Chou PC Chu HY. Clinical Efficacy of Acupuncture on Rheumatoid Arthritis and Associated Mechanisms: A Systemic Review. Evid Based Complement Alternat Med (2018) 2018:8596918. doi: 10.1155/2018/8596918 29849731 PMC5925010

[14] Ding X Huang S Tang Y Lin J. Effectiveness and Safety of Ear Acupuncture for Allergic Rhinitis: A Protocol of Randomized Controlled Trial. Med (Baltimore) (2021) 100:e24943. doi: 10.1097/MD.0000000000024943 PMC9282036 33761651

[15] Jiao R Yang Z Wang Y Zhou J Zeng Y Liu Z. The Effectiveness and Safety of Acupuncture for Patients With Atopic Eczema: A Systematic Review and Meta-Analysis. Acupunct Med (2020) 38:3–14. doi: 10.1177/0964528419871058 31495184 PMC7041622

[16] Vickers AJ Vertosick EA Lewith G Macpherson H Foster NE Sherman KJ. Acupuncture for Chronic Pain: Update of an Individual Patient Data Meta-Analysis. J Pain (2018) 19:455–74. doi: 10.1016/j.jpain.2017.11.005 PMC5927830 29198932

[17] Dimitrova A Murchison C Oken B. Acupuncture for the Treatment of Peripheral Neuropathy: A Systematic Review and Meta-Analysis. J Altern Complement Med (2017) 23:164–79. doi: 10.1089/acm.2016.0155 PMC5359694 28112552

[18] Torres-Rosas R Yehia G Pena G Mishra P Del Rocio Thompson-Bonilla M Moreno-Eutimio MA. Dopamine Mediates Vagal Modulation of the Immune System by Electroacupuncture. Nat Med (2014) 20:291–5. doi: 10.1038/nm.3479 PMC3949155 24562381

[19] W.H.O. WHO Standard Acupuncture Point Locations in the Western Pacific Region. In: WHO Standard Acupuncture Point Locations in the Western Pacific Region. Geneva, Switzerland: World Health Organization (2008).

[20] Zhu SF Guo H Zhang RR Zhang Y Li J Zhao XL. Effect of Electroacupuncture on the Inflammatory Response in Patients With Acute Pancreatitis: An Exploratory Study. Acupunct Med (2015) 33:115–20. doi: 10.1136/acupmed-2014-010646 25520280

[21] Wang LR Cai LY Lin DS Cao B Li ZJ. Effect of Electroacupuncture at The Zusanli Point (Stomach-36) on Dorsal Random Pattern Skin Flap Survival in a Rat Model. Dermatol Surg (2017) 43:1213–20. doi: 10.1097/DSS.0000000000001178 28445199

[22] Zhang Z Yu Q Zhang X Wang X Su Y He W. Electroacupuncture Regulates Inflammatory Cytokines by Activating the Vagus Nerve to Enhance Antitumor Immunity in Mice With Breast Tumors. Life Sci (2021) 272:119259. doi: 10.1016/j.lfs.2021.119259 33636172

[23] Huang W Long W Xiao J Zhao G Yu T. Effect of Electrically Stimulating Acupoint, Zusanli (ST 36), on Patient's Recovery After Laparoscopic Colorectal Cancer Resection: A Randomized Controlled Trial. J Tradit Chin Med (2019) 39:433–9. 32186016

[24] Liu S Wang ZF Su YS Ray RS Jing XH Wang YQ. Somatotopic Organization and Intensity Dependence in Driving Distinct NPY-Expressing Sympathetic Pathways by Electroacupuncture. Neuron (2020) 108:436–450 e437. doi: 10.1016/j.neuron.2020.07.015 32791039 PMC7666081

[25] Liu S Wang Z Su Y Qi L Yang W Fu M. A Neuroanatomical Basis for Electroacupuncture to Drive the Vagal-Adrenal Axis. Nature (2021) 598:641–5. doi: 10.1038/s41586-021-04001-4 PMC9178665 34646018

[26] Lai F Ren Y Lai C Chen R Yin X Tan C. Acupuncture at Zusanli (ST36) for Experimental Sepsis: A Systematic Review. Evid Based Complement Alternat Med (2020) 2020:3620741. doi: 10.1155/2020/3620741 32215037 PMC7081026

[27] Zhang L Wu Z Zhou J Lu S Wang C Xia Y. Electroacupuncture Ameliorates Acute Pancreatitis: A Role for the Vagus Nerve-Mediated Cholinergic Anti-Inflammatory Pathway. Front Mol Biosci (2021) 8:647647. doi: 10.3389/fmolb.2021.647647 34055878 PMC8155617

[28] Li Y Xu G Hu S Wu H Dai Y Zhang W. Electroacupuncture Alleviates Intestinal Inflammation and Barrier Dysfunction by Activating Dopamine in a Rat Model of Intestinal Ischaemia. Acupunct Med (2021) 39:208–16. doi: 10.1177/0964528420922232 32517478

[29] Xie DP Zhou GB Chen RL Qin XL Du JD Zhang Y. Effect of Electroacupuncture at Zusanli (ST36) on Sepsis Induced by Cecal Ligation Puncture and Its Relevance to Spleen. Evid Based Complement Alternat Med (2020) 2020:1914031. doi: 10.1155/2020/1914031 33082818 PMC7563055

[30] Liu GH Liu HM Chen YS Lee TY. Effect of Electroacupuncture in Mice With Dextran Sulfate Sodium-Induced Colitis and the Influence of Gut Microbiota. Evid Based Complement Alternat Med (2020) 2020:2087903. doi: 10.1155/2020/2087903 32419794 PMC7204379

[31] Lim HD Kim KJ Jo BG Park JY Namgung U. Acupuncture Stimulation Attenuates TNF-Alpha Production *via* Vagal Modulation in the Concanavalin A Model of Hepatitis. Acupunct Med (2020) 38:417–25. doi: 10.1177/0964528420907338 32233774

[32] Xie LL Zhao YL Yang J Cheng H Zhong ZD Liu YR. Electroacupuncture Prevents Osteoarthritis of High-Fat Diet-Induced Obese Rats. BioMed Res Int (2020) 2020:9380965. doi: 10.1155/2020/9380965 32724821 PMC7366230

[33] Wang L An J Song S Mei M Li W Ding F. Electroacupuncture Preserves Intestinal Barrier Integrity Through Modulating the Gut Microbiota in DSS-Induced Chronic Colitis. Life Sci (2020) 261:118473. doi: 10.1016/j.lfs.2020.118473 32971101

[34] Yang NN Ye Y Tian ZX Ma SM Zheng Y Huang J. Effects of Electroacupuncture on the Intestinal Motility and Local Inflammation Are Modulated by Acupoint Selection and Stimulation Frequency in Postoperative Ileus Mice. Neurogastroenterol Motil (2020) 32:e13808. doi: 10.1111/nmo.13808 32114712

[35] Zhao YX Yao MJ Liu Q Xin JJ Gao JH Yu XC. Electroacupuncture Treatment Attenuates Paclitaxel-Induced Neuropathic Pain in Rats *via* Inhibiting Spinal Glia and the TLR4/NF-kappaB Pathway. J Pain Res (2020) 13:239–50. doi: 10.2147/JPR.S241101 PMC7005725 32099448

[36] Harpin D Simadibrata CL Mihardja H Barasila AC. Effect of Electroacupuncture on Urea and Creatinine Levels in the Wistar Sepsis Model. Med Acupunct (2020) 32:29–37. doi: 10.1089/acu.2019.1369 32104525 PMC7041329

[37] Jin H Guo J Liu J Lyu B Foreman RD Shi Z. Autonomically Mediated Anti-Inflammatory Effects of Electrical Stimulation at Acupoints in a Rodent Model of Colonic Inflammation. Neurogastroenterol Motil (2019) 31:e13615. doi: 10.1111/nmo.13615 31117153

[38] Song S An J Li Y Liu S. Electroacupuncture at ST-36 Ameliorates DSS-Induced Acute Colitis *via* Regulating Macrophage Polarization Induced by Suppressing NLRP3/IL-1beta and Promoting Nrf2/HO-1. Mol Immunol (2019) 106:143–52. doi: 10.1016/j.molimm.2018.12.023 30610999

[39] Wang Z Lu M Ren J Wu X Long M Chen L. Electroacupuncture Inhibits Mast Cell Degranulation *via* Cannabinoid CB2 Receptors in a Rat Model of Allergic Contact Dermatitis. Acupunct Med (2019) 37:348–55. doi: 10.1136/acupmed-2017-011506 31429590

[40] Chen T Xiong Y Long M Zheng D Ke H Xie J. Electro-Acupuncture Pretreatment at Zusanli (ST36) Acupoint Attenuates Lipopolysaccharide-Induced Inflammation in Rats by Inhibiting Ca(2+) Influx Associated With Cannabinoid CB2 Receptors. Inflammation (2019) 42:211–20. doi: 10.1007/s10753-018-0885-5 30168040

[41] Zhang K Guo XM Yan YW Liu YY Xu ZF Zhao X. Applying Statistical and Complex Network Methods to Explore the Key Signaling Molecules of Acupuncture Regulating Neuroendocrine-Immune Network. Evid Based Complement Alternat Med (2018) 2018:9260630. doi: 10.1155/2018/9260630 29785197 PMC5896411

[42] Tian L Song S Zhu B Liu S. Electroacupuncture at ST-36 Protects Interstitial Cells of Cajal *via* Sustaining Heme Oxygenase-1 Positive M2 Macrophages in the Stomach of Diabetic Mice. Oxid Med Cell Longev (2018) 2018:3987134. doi: 10.1155/2018/3987134 29854081 PMC5944261

[43] Zhang L Huang Z Shi X Hu S Litscher D Wang L. Protective Effect of Electroacupuncture at Zusanli on Myocardial Injury in Septic Rats. Evid Based Complement Alternat Med (2018) 2018:6509650. doi: 10.1155/2018/6509650 30402132 PMC6196882

[44] Chen L Xu A Yin N Zhao M Wang Z Chen T. Enhancement of Immune Cytokines and Splenic CD4+ T Cells by Electroacupuncture at ST36 Acupoint of SD Rats. PloS One (2017) 12:e0175568. doi: 10.1371/journal.pone.0175568 28406959 PMC5391063

[45] Geng Y Chen D Zhou J Jiang H Zhang H. Role of Cholinergic Anti-Inflammatory Pathway in Treatment of Intestinal Ischemia-Reperfusion Injury by Electroacupuncture at Zusanli. Evid Based Complement Alternat Med (2017) 2017:6471984. doi: 10.1155/2017/6471984 29333186 PMC5733189

[46] Wang Z Yi T Long M Gao Y Cao C Huang C. Electro-Acupuncture at Zusanli Acupoint (ST36) Suppresses Inflammation in Allergic Contact Dermatitis *via* Triggering Local IL-10 Production and Inhibiting P38 MAPK Activation. Inflammation (2017) 40:1351–64. doi: 10.1007/s10753-017-0578-5 28493082

[47] Wang Z Chen T Long M Chen L Wang L Yin N. Electro-Acupuncture at Acupoint ST36 Ameliorates Inflammation and Regulates Th1/Th2 Balance in Delayed-Type Hypersensitivity. Inflammation (2017) 40:422–34. doi: 10.1007/s10753-016-0487-z 27966183

[48] Lim HD Kim MH Lee CY Namgung U. Anti-Inflammatory Effects of Acupuncture Stimulation *via* the Vagus Nerve. PloS One (2016) 11:e0151882. doi: 10.1371/journal.pone.0151882 26991319 PMC4798687

[49] Liu M Zhang S Gai Y Xie M Qi Q. Changes in the Interstitial Cells of Cajal and Immunity in Chronic Psychological Stress Rats and Therapeutic Effects of Acupuncture at the Zusanli Point (St36). Evid Based Complement Alternat Med (2016) 2016:1935372. doi: 10.1155/2016/1935372 27594888 PMC4987473

[50] Geng Y Chen D Zhou J Lu J Chen M Zhang H. Synergistic Effects of Electroacupuncture and Mesenchymal Stem Cells on Intestinal Ischemia/Reperfusion Injury in Rats. Inflammation (2016) 39:1414–20. doi: 10.1007/s10753-016-0373-8 27221138

[51] Wen CK Lee TY. Electroacupuncture Prevents White Adipose Tissue Inflammation Through Modulation of Hypoxia-Inducible Factors-1alpha-Dependent Pathway in Obese Mice. BMC Complement Altern Med (2015) 15:452. doi: 10.1186/s12906-015-0977-9 26714835 PMC4696133

[52] Zhu MF Xing X Lei S Wu JN Wang LC Huang LQ. Electroacupuncture at Bilateral Zusanli Points (ST36) Protects Intestinal Mucosal Immune Barrier in Sepsis. Evid Based Complement Alternat Med (2015) 2015:639412. doi: 10.1155/2015/639412 26346309 PMC4539462

[53] Hu S Zhao ZK Liu R Wang HB Gu CY Luo HM. Electroacupuncture Activates Enteric Glial Cells and Protects the Gut Barrier in Hemorrhaged Rats. World J Gastroenterol (2015) 21:1468–78. doi: 10.3748/wjg.v21.i5.1468 PMC4316089 25663766

[54] Song XM Wu XJ Li JG Le LL Liang H Xu Y. The Effect of Electroacupuncture at ST36 on Severe Thermal Injury-Induced Remote Acute Lung Injury in Rats. Burns (2015) 41:1449–58. doi: 10.1016/j.burns.2015.03.004 26188895

[55] Villegas-Bastida A Torres-Rosas R Arriaga-Pizano LA Flores-Estrada J Gustavo-Acosta A Moreno-Eutimio MA. Electrical Stimulation at the ST36 Acupoint Protects Against Sepsis Lethality and Reduces Serum TNF Levels Through Vagus Nerve- and Catecholamine-Dependent Mechanisms. Evid Based Complement Alternat Med (2014) 2014:451674. doi: 10.1155/2014/451674 25057275 PMC4098981

[56] Wen CK Lee TY. Electroacupuncture Decreases the Leukocyte Infiltration to White Adipose Tissue and Attenuates Inflammatory Response in High Fat Diet-Induced Obesity Rats. Evid Based Complement Alternat Med (2014) 2014:473978. doi: 10.1155/2014/473978 25202333 PMC4150518

[57] Peng MF Li K Wang C Zhu XY Yang Z Zhang GH. Therapeutic Effect and Mechanism of Electroacupuncture at Zusanli on Plasticity of Interstitial Cells of Cajal: A Study of Rat Ileum. BMC Complement Altern Med (2014) 14:186. doi: 10.1186/1472-6882-14-186 24908398 PMC4096531

[58] Xue QM Li N Xue P Wang CW Wen Q. Therapeutic Effects of Electroacupuncture at ST36 Acupoint on Sodium-Taurocholate-Induced Severe Acute Pancreatitis. Chin J Integr Med (2014) 20:695–700. doi: 10.1007/s11655-013-1331-4 23893236

[59] Song Q Hu S Wang H Lv Y Shi X Sheng Z. Electroacupuncturing at Zusanli Point (ST36) Attenuates Pro-Inflammatory Cytokine Release and Organ Dysfunction by Activating Cholinergic Anti-Inflammatory Pathway in Rat With Endotoxin Challenge. Afr J Tradit Complement Altern Med (2014) 11:469–74. doi: 10.4314/ajtcam.v11i2.35 PMC4202659 25435635

[60] Du MH Luo HM Hu S Lv Y Lin ZL Ma L. Electroacupuncture Improves Gut Barrier Dysfunction in Prolonged Hemorrhagic Shock Rats Through Vagus Anti-Inflammatory Mechanism. World J Gastroenterol (2013) 19:5988–99. doi: 10.3748/wjg.v19.i36.5988 PMC3785620 24106399

[61] Hu S Du MH Luo HM Wang H Lv Y Ma L. Electroacupuncture at Zusanli (ST36) Prevents Intestinal Barrier and Remote Organ Dysfunction Following Gut Ischemia Through Activating the Cholinergic Anti-Inflammatory-Dependent Mechanism. Evid Based Complement Alternat Med (2013) 2013:592127. doi: 10.1155/2013/592127 23662144 PMC3638586

[62] Geng WY Liu ZB Song NN Geng WY Zhang GH Jin WZ. Effects of Electroacupuncture at Zusanli (ST36) on Inflammatory Cytokines in a Rat Model of Smoke-Induced Chronic Obstructive Pulmonary Disease. J Integr Med (2013) 11:213–9. doi: 10.3736/jintegrmed2013024 23743164

[63] Gim GT Lee JH Park E Sung YH Kim CJ Hwang WW. Electroacupuncture Attenuates Mechanical and Warm Allodynia Through Suppression of Spinal Glial Activation in a Rat Model of Neuropathic Pain. Brain Res Bull (2011) 86:403–11. doi: 10.1016/j.brainresbull.2011.09.010 21958939

[64] Liu YM Liu XJ Bai SS Mu LL Kong QF Sun B. The Effect of Electroacupuncture on T Cell Responses in Rats With Experimental Autoimmune Encephalitis. J Neuroimmunol (2010) 220:25–33. doi: 10.1016/j.jneuroim.2009.12.005 20117842

[65] An HJ Lee JH Lee HJ Yang WM Park SK Hong SH. Electroacupuncture Protects Against CCK-Induced Acute Pancreatitis in Rats. Neuroimmunomodulation (2007) 14:112–8. doi: 10.1159/000107793 17804915

[66] Chae Y Hong MS Kim GH Hahm DH Park HJ Ha E. Protein Array Analysis of Cytokine Levels on the Action of Acupuncture in Carrageenan-Induced Inflammation. Neurol Res (2007) 29(Suppl 1):S55–58. doi: 10.1179/016164107X172365 17359642

[67] Yim YK Lee H Hong KE Kim YI Lee BR Son CG. Electro-Acupuncture at Acupoint ST36 Reduces Inflammation and Regulates Immune Activity in Collagen-Induced Arthritic Mice. Evid Based Complement Alternat Med (2007) 4:51–7. doi: 10.1093/ecam/nel054 PMC1810363 17342241

[68] Tian L Huang YX Tian M Gao W Chang Q. Downregulation of Electroacupuncture at ST36 on TNF-Alpha in Rats With Ulcerative Colitis. World J Gastroenterol (2003) 9:1028–33. doi: 10.3748/wjg.v9.i5.1028 PMC4611366 12717850

[69] Chen Y Cheng J Zhang Y Chen JDZ Seralu FM. Electroacupuncture at ST36 Relieves Visceral Hypersensitivity *via* the NGF/TrkA/TRPV1 Peripheral Afferent Pathway in a Rodent Model of Post-Inflammation Rectal Hypersensitivity. J Inflamm Res (2021) 14:325–39. doi: 10.2147/JIR.S285146 PMC7875081 33584100

[70] Wang Z Yi T Long M Ding F Ouyang L Chen Z. Involvement of the Negative Feedback of IL-33 Signaling in the Anti-Inflammatory Effect of Electro-Acupuncture on Allergic Contact Dermatitis *via* Targeting MicroRNA-155 in Mast Cells. Inflammation (2018) 41:859–69. doi: 10.1007/s10753-018-0740-8 29404871

[71] Du MH Luo HM Tian YJ Zhang LJ Zhao ZK Lv Y. Electroacupuncture ST36 Prevents Postoperative Intra-Abdominal Adhesions Formation. J Surg Res (2015) 195:89–98. doi: 10.1016/j.jss.2014.12.043 25619463

[72] Goes AC Pinto FM Fernandes GC Barbosa JS Correia ES Ribeiro RA. Electroacupuncture Ameliorates Experimental Colitis Induced by TNBS Through Activation of Interleukin-10 and Inhibition of iNOS in Mice. Acta Cir Bras (2014) 29:787–93. doi: 10.1590/S0102-86502014001900004 25517491

[73] Zhang L Wang H Huang Z Shi X Hu S Gaischek I. Inhibiting Effect of Electroacupuncture at Zusanli on Early Inflammatory Factor Levels Formed by Postoperative Abdominal Adhesions. Evid Based Complement Alternat Med (2014) 2014:950326. doi: 10.1155/2014/950326 25197314 PMC4145794

[74] Aguiar DN Silva MM Parreira WV Tome FD Batista LF Gomes CM. Electroacupuncture at the ST36 Acupoint Increases Interleukin-4 Responsiveness in Macrophages, Generation of Alternatively Activated Macrophages and Susceptibility to Leishmania Major Infection. Chin Med (2012) 7:17. doi: 10.1186/1749-8546-7-17 22838729 PMC3444353

[75] Xu X Li Q Zhou L Ru L. Neurochemical Mechanism of the Gastrointestinal Interdigestive Migrating Motor Complex in Rats With Acute Inflammatory Stomach Ache. Neural Regener Res (2012) 7:2136–43. doi: 10.3969/j.issn.1673-5374.2012.27.008 PMC4281416 25558227

[76] Zhao P Chen X Han X Wang Y Shi Y Ji J. Involvement of microRNA-155 in the Mechanism of Electroacupuncture Treatment Effects on Experimental Autoimmune Encephalomyelitis. Int Immunopharmacol (2021) 97:107811. doi: 10.1016/j.intimp.2021.107811 34091117

[77] Li Y Fang Z Gu N Bai F Ma Y Dong H. Inhibition of Chemokine CX3CL1 in Spinal Cord Mediates the Electroacupuncture-Induced Suppression of Inflammatory Pain. J Pain Res (2019) 12:2663–72. doi: 10.2147/JPR.S205987 PMC6732508 31564958

[78] Kim S Zhang X O'buckley SC Cooter M Park JJ Nackley AG. Acupuncture Resolves Persistent Pain and Neuroinflammation in a Mouse Model of Chronic Overlapping Pain Conditions. J Pain (2018) 19(1384):e1381–1384 e1314. doi: 10.1016/j.jpain.2018.05.013 PMC6289709 29981376

[79] Huang CP Chen HN Su HL Hsieh CL Chen WH Lai ZR. Electroacupuncture Reduces Carrageenan- and CFA-Induced Inflammatory Pain Accompanied by Changing the Expression of Nav1.7 and Nav1.8, Rather Than Nav1.9, in Mice Dorsal Root Ganglia. Evid Based Complement Alternat Med (2013) 2013:312184. doi: 10.1155/2013/312184 23573123 PMC3615619

[80] Chen WH Tzen JT Hsieh CL Chen YH Lin TJ Chen SY. Attenuation of TRPV1 and TRPV4 Expression and Function in Mouse Inflammatory Pain Models Using Electroacupuncture. Evid Based Complement Alternat Med (2012) 2012:636848. doi: 10.1155/2012/636848 23258994 PMC3520481

[81] Zhang Z Wang C Gu G Li H Zhao H Wang K. The Effects of Electroacupuncture at the ST36 (Zusanli) Acupoint on Cancer Pain and Transient Receptor Potential Vanilloid Subfamily 1 Expression in Walker 256 Tumor-Bearing Rats. Anesth Analg (2012) 114:879–85. doi: 10.1213/ANE.0b013e318246536d 22253272

[82] Chen WH Hsieh CL Huang CP Lin TJ Tzen JT Ho TY. Acid-Sensing Ion Channel 3 Mediates Peripheral Anti-Hyperalgesia Effects of Acupuncture in Mice Inflammatory Pain. J BioMed Sci (2011) 18:82. doi: 10.1186/1423-0127-18-82 22070775 PMC3233511

[83] Yang EJ Jiang JH Lee SM Hwang HS Lee MS Choi SM. Electroacupuncture Reduces Neuroinflammatory Responses in Symptomatic Amyotrophic Lateral Sclerosis Model. J Neuroimmunol (2010) 223:84–91. doi: 10.1016/j.jneuroim.2010.04.005 20460191

[84] Kim HW Kang SY Yoon SY Roh DH Kwon YB Han HJ. Low-Frequency Electroacupuncture Suppresses Zymosan-Induced Peripheral Inflammation *via* Activation of Sympathetic Post-Ganglionic Neurons. Brain Res (2007) 1148:69–75. doi: 10.1016/j.brainres.2007.02.030 17367766

[85] Sung HJ Kim YS Kim IS Jang SW Kim YR Na DS. Proteomic Analysis of Differential Protein Expression in Neuropathic Pain and Electroacupuncture Treatment Models. Proteomics (2004) 4:2805–13. doi: 10.1002/pmic.200300821 15352254

[86] Yang F Gong Y Yu N Yao L Zhao X Hong S. ST36 Acupuncture Alleviates the Inflammation of Adjuvant-Induced Arthritic Rats by Targeting Monocyte/Macrophage Modulation. Evid Based Complement Alternat Med (2021) 2021:9430501. doi: 10.1155/2021/9430501 33727948 PMC7936911

[87] Zhang K Zhao X Ding S Liu Y Xu Y Yan Y. Applying Complex Network and Cell-Cell Communication Network Diagram Methods to Explore the Key Cytokines and Immune Cells in Local Acupoint Involved in Acupuncture Treating Inflammatory Pain. Evid Based Complement Alternat Med (2020) 2020:2585960. doi: 10.1155/2020/2585960 32802117 PMC7411476

[88] Li H Shang XJ Dong QR. Effects of Transcutaneous Electrical Nerve Stimulation on Rats With the Third Lumbar Vertebrae Transverse Process Syndrome. Acupunct Med (2015) 33:400–5. doi: 10.1136/acupmed-2014-010752 26104377

[89] Wu SY Chen WH Hsieh CL Lin YW. Abundant Expression and Functional Participation of TRPV1 at Zusanli Acupoint (ST36) in Mice: Mechanosensitive TRPV1 as an "Acupuncture-Responding Channel". BMC Complement Altern Med (2014) 14:96. doi: 10.1186/1472-6882-14-96 24612851 PMC3984709

[90] Smeester BA Al-Gizawiy M Beitz AJ. Effects of Different Electroacupuncture Scheduling Regimens on Murine Bone Tumor-Induced Hyperalgesia: Sex Differences and Role of Inflammation. Evid Based Complement Alternat Med (2012) 2012:671386. doi: 10.1155/2012/671386 23320035 PMC3541553

[91] Jiang JH Yang EJ Baek MG Kim SH Lee SM Choi SM. Anti-Inflammatory Effects of Electroacupuncture in the Respiratory System of a Symptomatic Amyotrophic Lateral Sclerosis Animal Model. Neurodegener Dis (2011) 8:504–14. doi: 10.1159/000327911 21849797

[92] Kim HW Uh DK Yoon SY Roh DH Kwon YB Han HJ. Low-Frequency Electroacupuncture Suppresses Carrageenan-Induced Paw Inflammation in Mice *via* Sympathetic Post-Ganglionic Neurons, While High-Frequency EA Suppression Is Mediated by the Sympathoadrenal Medullary Axis. Brain Res Bull (2008) 75:698–705. doi: 10.1016/j.brainresbull.2007.11.015 18355649

[93] Moon PD Jeong HJ Kim SJ An HJ Lee HJ Yang WM. Use of Electroacupuncture at ST36 to Inhibit Anaphylactic and Inflammatory Reaction in Mice. Neuroimmunomodulation (2007) 14:24–31. doi: 10.1159/000107285 17700037

